# Cadavers stored under refrigerated conditions as a source of cold-adapted microscopic fungi

**DOI:** 10.1007/s00253-026-13958-z

**Published:** 2026-07-11

**Authors:** Klaudyna Spychała, Agata Piecuch, Łukasz Szleszkowski, Agata Thannhäuser, Marcin Kadej, Rafał Ogórek

**Affiliations:** 1 Department of Mycology and Genetics, Faculty of Biological Sciences, University of Wrocław, Przybyszewskiego 63, Wroclaw, 51-148 Poland; 2 Department of Forensic Medicine, Wroclaw Medical University, Mikulicza-Radeckiego 4, Wrocław, 50-345 Poland; 3 Centre for Forensic Biology and Entomology, Department of Invertebrate Biology, Evolution and Conservation, Faculty of Biological Sciences, University of Wrocław, Przybyszewskiego 65, Wrocław, 51-148 Poland

**Keywords:** Fungi, Cadaver, Cold-adapted fungi, Refrigerated conditions, Diversity

## Abstract

**Abstract:**

Fungi are frequently observed on human remains during decomposition; however, little is known about fungal communities associated with cadavers stored under refrigerated conditions. This study aimed to characterize the cultivable fungal diversity of human cadavers subjected to long-term cold storage and to identify taxa capable of persisting under these conditions. A total of 12 cadavers were examined, including refrigerated cadavers stored at 4 °C, frozen cadavers, and one non-refrigerated comparison case recovered from an indoor environment. Fungi were isolated using culture-based methods and identified using morphological and molecular approaches. The influence of four culture media (Yeast Peptone Glucose – YPG, Potato Dextrose Agar – PDA, Sabouraud agar with glucose – SGA, and Modified Leeming–Notman agar – MLNA) and three incubation temperatures (5, 24, and 37 °C) on fungal recovery was also evaluated. In total, 270 fungal isolates representing 73 species were obtained. The most frequently isolated species were *Yarrowia lipolytica*, *Penicillium crustosum*, *Debaryomyces hansenii*, *Penicillium polonicum*, and *Pseudogymnoascus pannorum*. PDA medium and incubation at 24 °C yielded the highest species richness. A negative correlation was observed between fungal species richness and storage time in a cold room. Several fungal taxa were recorded for the first time from human cadavers. These findings provide new baseline data on fungi associated with cold-stored human remains and contribute to the development of forensic and environmental mycology.

**Key points:**

• *Fungal species recovered from refrigerated human cadavers.*

• *Penicillium and yeast species dominated the fungal assemblages.*

• *Species richness decreased with increasing storage time.*

**Graphical Abstract:**

**Figure:**
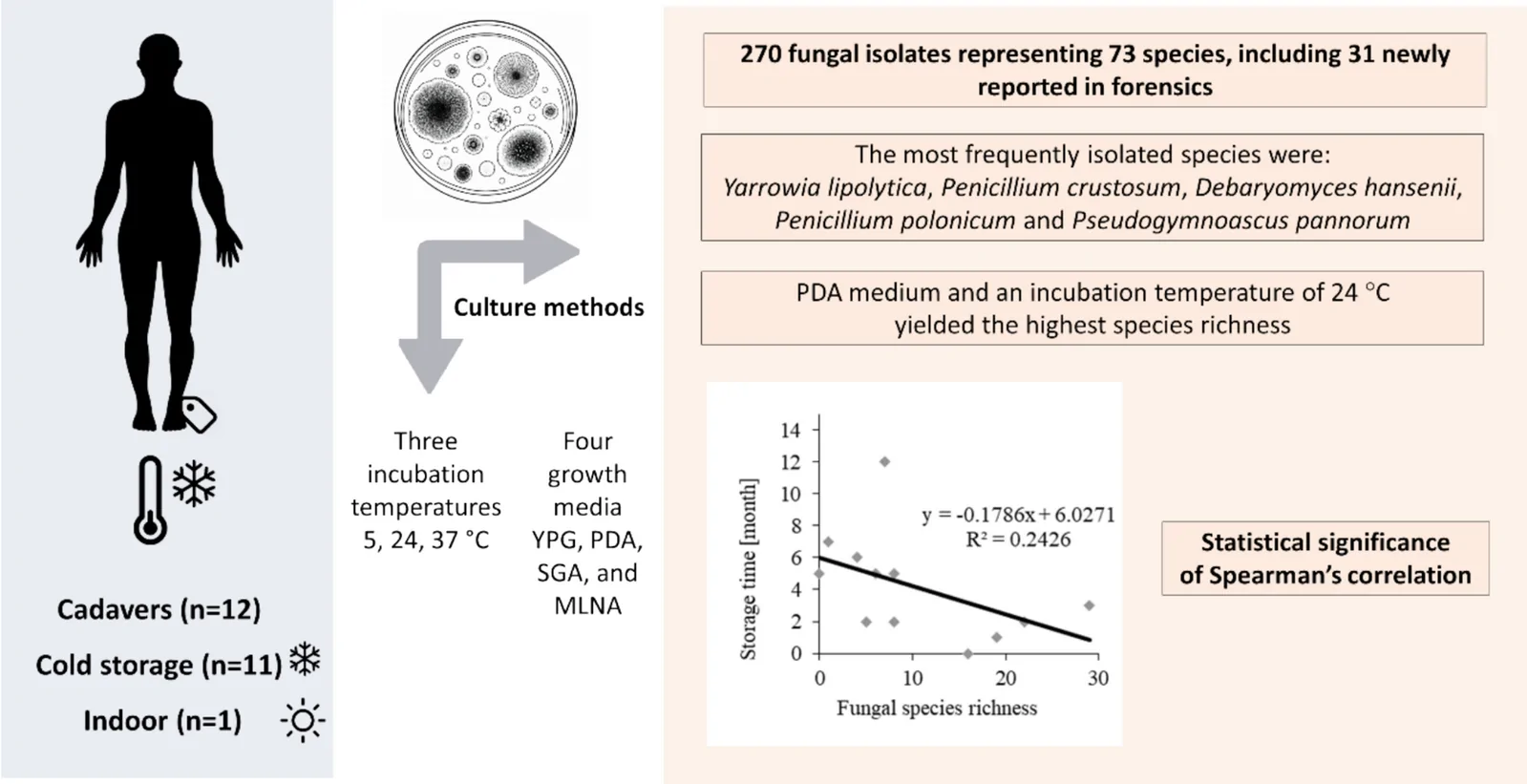

**Supplementary Information:**

The online version contains supplementary material available at https://doi.org/10.1007/s00253-026-13958-z.

## Introduction

Fungi are important decomposers capable of colonizing a wide range of organic substrates, including human remains. Their development on cadavers is facilitated by the abundance of nutrients and the absence of an immune response after death (Janaway et al. 2009). Fungi associated with corpses may originate from both outdoor and indoor environments, as fungal propagules are ubiquitous in air. Consequently, fungal growth and colonization can occur not only on remains exposed to natural environmental conditions but also on cadavers stored in controlled indoor settings, such as morgues and autopsy rooms (Schwarz et al. 2015; personal observations).

Previous studies have shown that microscopic fungi may become visible on cadavers within a few days postmortem and can contribute to tissue degradation through enzymatic activity and structural penetration of bones (Janaway et al. 2009; Spychała et al. 2024). Importantly, microorganisms, including fungi, are capable of forming biofilms on the body surface, interact with one another in complex ways, and many of them are capable of producing antibiotic substances, thereby influencing the postmortem microbial ecosystem (Janaway et al. 2009). As a result, fungal communities constitute an important yet often overlooked component of the postmortem microbiota.

Temperature is one of the most important environmental factors shaping fungal growth, survival, and community composition. Although low temperatures generally slow biological processes, numerous fungal taxa are capable of growth or long-term survival under refrigerated conditions. Psychrophilic and psychrotolerant species, in particular, remain active or metabolically flexible under cold conditions, and are commonly encountered in cold natural and artificial environments (Morita 1975; Wang et al. 2017). Consequently, cadavers stored in cold rooms—typically at 4 °C—may still undergo microbial and fungal colonization despite reduced decomposition rates. Fungal growth has been repeatedly observed on bodies kept in refrigeration for extended periods, including cases stored before or after autopsy (Schwarz et al. 2015).

The study of fungi associated with refrigerated human remains is relevant for several reasons. First, refrigerated storage is routinely used in forensic and hospital practice, and bodies may remain in cold rooms for weeks or even months before burial or further examination. Second, fungal colonization may affect the condition of tissues and potentially influence postmortem changes observed during forensic investigations (Janaway et al. 2009). Finally, personnel handling refrigerated remains may be exposed to fungal species with pathogenic potential (Schwarz et al. 2015). Despite these considerations, research on fungi associated with refrigerated cadavers remains limited.

Despite extensive research on postmortem bacterial communities, the fungal species composition of cadavers stored under refrigerated conditions remains largely unexplored. To date, only a few studies have investigated fungal succession or species composition on cold-stored human remains, and comprehensive analyses of fungal diversity under these conditions are scarce (Di Piazza et al. 2018). Furthermore, little is known about the occurrence of fungi on refrigerated bodies lacking macroscopically visible fungal growth, despite the possibility that viable fungal propagules may still be present.

Therefore, the aim of this study was to characterize the culturable fungal communities associated with human cadavers stored under refrigerated conditions for at least one month and to identify cold-adapted taxa occurring in this environment. By combining culture-based methods, multi-temperature incubation, and molecular identification, we provide one of the most comprehensive analyses to date of the composition of fungal communities associated with refrigerated human remains to date and contributes to the growing field of forensic and environmental mycology.

## Materials and methods

### Characteristics of the study material

The study included 12 cadavers. Nine cadavers had been stored in a mortuary cold room at 4 °C for at least one month and showed visible macroscopic fungal growth. Of these, two were subsequently transferred to a freezer maintained at − 10 °C after several months of refrigerated storage to further limit decomposition processes. The fungi isolated from the frozen cadavers most likely originated from colonization that occurred during the preceding storage period at 4 °C and remained viable after freezing. Two additional frozen cadavers without visible fungal growth (cadavers IV and V) were included for comparison. Furthermore, one non-refrigerated cadaver recovered from a residential indoor environment, in a state of decomposition with signs of mummification (cadaver XII) was examined as an additional comparison case. Detailed data, including the approximate duration of storage in the cold room, the condition of the bodies at the time of autopsy (prior to their placement in refrigerated conditions), and the environments in which the bodies were found, are presented in Table 1. Information on the decomposition stage was obtained from forensic pathology records and was based on the macroscopic assessment of the cadavers performed by a forensic pathologist**.** In none of the cases was a fungal infection found, although several were hospitalized before death.

**Table 1:** Characteristics of the cadavers included in the study and the diversity of fungi isolated from individual cases

Case number	Sex	Approximate refrigerated storage time	Frozen/refrigerated	Stage of a cadaver decomposition (during an autopsy)	Hospitalization	Environment of finding the cadaver	Visible fungal growth	Number of fungi		
								Isolates	Species	Genera
I	Male	One year	Frozen	Fresh stage	No	Outdoor, bus stop, urban area, autumn	Yes	22	7	5
II	Female	Seven months	Refrigerated	Fresh stage	Yes	Indoor, hospital,urban area, winter	Yes	1	1	1
III	Male	Six months	Refrigerated	Fresh stage	No	Outdoor, roadside, rural, spring	Yes	13	4	3
IV	Female	Five months	Frozen	Fresh stage	No	Indoor, house, urban area, summer	No **(control)**	0	0	0
V	Male	Five months	Frozen	Advanced decay	No	Indoor, house, urban area, summer	No **(control)**	25	6	4
VI	Male	Five months	Refrigerated	Fresh stage	No	Outdoor, roadside, urban area, summer	Yes	11	8	4
VII	Male	Three months	Refrigerated	Advanced decay	No	Outdoor, forest, wetland, summer	Yes	56	29	23
VIII	Male	Two months	Refrigerated	Fresh stage	No	Outdoor, car park, urban area, summer	Yes	14	8	5
IX	Female	Two months	Refrigerated	Fresh stage	Yes	Indoor, hospital, urban area, summer	Yes	15	5	5
X	Male	One month	Refrigerated	Fresh stage	No	Indoor, house, urban area, autumn	Yes	29	19	11
XI	Male	Two months (in the hospital cold storage)	Frozen	Advanced postmortem changes	Yes	Indoor, hospital, urban area, autumn	Yes	60	22	10
XII	Male	Not stored in a cold store	No	Decay with signs of mummification	No	Indoor, house, urban area, autumn	Yes	24	16	10

### Sample collection

Samples were taken from the corpses on November 17, 2022, and November 24, 2022, at the Department of Forensic Medicine of the Wroclaw Medical University, in accordance with standard microbiological procedures, i.e., using a viscose swab moistened with saline solution (0.85% NaCl) (Spychała et al. 2024). The material was collected from selected parts of the body. In the case of corpses with macroscopically visible mycelium, the focus was on visible colonies – corpses no. I, II, II, VI, VII, VIII, IX, X, XI, and XII (Table 1). Details of the sampling locations and the corresponding fungal species recovered from each anatomical site are provided in Table S1. Due to differences between sampling sites, anatomical location was not included as a comparative variable.

The samples were stored at 5 ± 0.5 °C in transport tubes (Spychała et al. 2024) until further analysis.

### Isolation of fungi from samples

The isolation of fungi from the cadavers was started by sterile cutting of the tips of the swabs with the collected material and placing them in sterile conical polypropylene tubes (25 ml) with screw caps (FL Medical, Torreglia, Italy) containing 10 ml of physiological saline solution (0.85% NaCl). This procedure was used to achieve homogeneous distribution of the material. The tubes were shaken at room temperature (20 min; 24 ± 1 °C), and then 100 µl of the sample was spread on plates with culture medium in three replicates for each incubation temperature (Spychała et al. 2025).

To capture the widest possible range of species, four culture media were used (Spychała et al. 2025):

- YPG medium (consisting of 20 g·L − 1 glucose, 20 g·L − 1 peptone, 20 g·L − 1 agar, 10 g·L − 1 yeast extract) with a pH reduced to 4.5 (YPG pH 4.5) containing chloramphenicol (50 mg·L − 1) to inhibit bacteria growth and certain filamentous fungi, according to Spychała et al. (2024);
- Potato dextrose agar (PDA) (Biomaxima, Lublin, Poland);
- Sabouraud agar with glucose (SGA) (consisting of 40 g·L − 1 glucose, 15 g·L − 1 agar, 10 g·L − 1 peptone);
- Modified Leeming–Notman agar (MLNA) (consisting of 1% glucose, 1% peptone, 1% glycerol, 2% olive oil, 1.5% agar, 0.8% bovine bile, 0.5% Tween 60, 0.2% yeast extract and 0.05% glycerol monostearate) for the isolation of *Malassezia* spp.

The material was incubated for 7 to 30 days at 3 temperatures (5 ± 0.5 °C, 24 ± 0.5 °C, and 37 ± 0.5 °C) to increase the yield of fungal communities. Pure cultures were obtained by the single spore method on PDA medium for filamentous fungi and YPD medium for yeast-like fungi. Isolates have stored on PDA slants under refrigerated conditions (filamentous fungi) and in glycerol at −70 ± 0.1 °C (yeast-like fungi) (Spychała et al. 2025).

### Fungal identification

The identification of fungi was carried out using a combination of classical and molecular methods. Phenotypic identification was conducted for all isolates grown on PDA medium. Additionally, representatives of the genera *Penicillium* and *Aspergillus* were cultivated on Czapek yeast autolysate agar (CYA; 30.0 g·L − 1 sucrose, 15.0 g·L − 1 agar, 5.0 g·L − 1 yeast extract, 3.0 g·L − 1 NaNO_3_, 1.0 g·L − 1 K_2_HPO_4_, 0.5 g·L − 1 KCl, 0.5 g·L − 1 MgSO_4_·7H_2_O, and 0.01 g·L − 1 FeSO_4_·7H_2_O) as well as malt extract agar (MEA; BioMaxima, Lublin, Poland). For filamentous fungi, identification was based on both macro- and micromorphological characteristics. Colony appearance, including texture, pigmentation of the obverse and reverse sides, colony diameter, and growth rate, was recorded. Microscopic observations focused on reproductive structures, such as the morphology and branching of conidiophores, together with the shape and dimensions of conidia or sporangia. Yeast isolates were characterized according to colony morphology and microscopic features, including cell morphology, budding pattern, and the presence of pseudohyphae or true hyphae. When relevant, physiological traits, such as growth at different temperatures, were also taken into account. Species identification was performed using standard taxonomic keys and mycological monographs (Barron 1962; Bensch et al. 2012; Boekhout et al. 1991; Cain 1961; Chen et al. 2015; Chuang et al. 2024; Crous et al. 2017; Gilgado et al. 2005; Gräfenhan et al. 2011; Kurtzman et al. 2011; Leslie and Summerell 2006; Luangsa-Ard et al. 2011; Middelhoven et al. 2004; Nagy et al. 2014; Samson et al. 2010; Sandoval-Denis et al. 2016; Schipper 1978; Sigler and Carmichael 1976; Sogonov et al. 2005; Stolk 1969; Stolk and Samson 1972; Thom 1910; Tian et al. 2024; Untereiner et al. 2019; van der Walt and von Arx 1980; Visagie et al. 2014; Visagie et al. 2019; Wang et al. 2019, 2022; Woudenberg et al. 2017; Zalar et al. 2007).

Molecular identification began with DNA isolation using the Bead-Beat Micro AX Gravity kit (A&A Biotechnology, Gdansk, Poland) performed according to the manufacturer's instructions. Polymerase chain reaction (PCR) was performed using a T100 thermocycler (Bio-Rad, Berkeley, California, USA) in accordance with the protocols of White et al. (1990) and Kurtzman and Robnett (1997) using one of two primer pairs:

- ITS1 (5′-TCCGTAGGTGAACCTGCGG-3′) and ITS4 (5′-TCCTCCGCTTATTGATGC-3′) for amplification of the internal transcribed spacer (ITS) region of fungal DNA (White et al. 1990), for filamentous fungi;
- NL-1 (5'-GCATATCAATAAGCGGAGGAAAAG-3') and NL-4 (5'-GGTCCGTGTTTCAAGACGG-3') for amplification of the D1/D2 region at the 5' end of the large subunit (LSU) rDNA gene (Kurtzman and Robnett 1997), for yeast-like fungi.

The PCR products were purified using a Clean-Up Kit (A&ABiotechnology, Gdansk, Poland) and then sequenced at Macrogen Europe (Amsterdam, Netherlands, https://www.macrogen-europe.com/).

### Data analyses

Sequence data were edited and evaluated using the BioEdit Sequence Alignment Editor (https://bioedit.software.informer.com/). For taxonomic assignment, PCR products were matched to reference sequences available in the GenBank database of the National Center for Biotechnology Information (NCBI, Bethesda, MD, USA) using the BLAST search tool. Confirmed sequences were then deposited in GenBank under accession numbers from PZ134796 to PZ134883 and from PZ134907 to PZ135088 (Table S2). All fungal strains were preserved in the culture collection of the Department of Mycology and Genetics at the University of Wroclaw.

Associations between fungal species richness and host traits (frozen/refrigerated, stage of a cadaver decomposition, hospitalization, visible fungal growth) were assessed using Spearman’s rank correlation coefficient (*r*_*S*_) at α = 0.05. For analyses, frozen/refrigerated was coded as 0 (not frozen); 1 (refrigerated) or 2 (frozen); stage of a cadaver decomposition was coded as 0 (fresh stage), 1 (advanced postmortem changes), 2 (advanced decay) or 3 (decay with signs of mummification); hospitalization was coded as 0 (no) or 1 (yes); and visible fungal growth was coded as 0 (no) or 1 (yes).

## Results

### Fungal diversity

Mycological analyses were performed separately for each cadaver, including refrigerated bodies stored at 4 °C, frozen cadavers, and one body recovered from a residential environment; however, all steps – from sampling to culture and identification – were carried out using identical procedures to ensure the reliability of the results. Owing to the variability in sampling sites between cases, anatomical location was not included as a comparative variable.

Based on differences in macro- and micromorphology, fungal isolates were divided into groups (isolates from UWR_900 to UWR_1169). One representative from each group was selected for genetic identification. Detailed data on the number of isolates and species isolated from individual cadavers are presented in Table 1. In summary, 270 fungal isolates belonging to 73 species and 25 families were obtained from all 12 cases studied (Table S2).

All fungal rDNA nucleotide sequences generated in this study are available in the GenBank database under accession numbers from PZ134796 to PZ134883 (yeast-like fungi sequenced using the NL-1/NL-4 primer) and from PZ134907 to PZ135088 (filamentous fungi identified using the ITS1/ITS4 primer pair). Based on BLAST analysis, the sequence lengths ranged from 351 to 583 bp and identity range of 99.20–100%. All sequences had an E-value of zero, and 100% query cover. Detailed information is provided in Table S2.

In order to analyze the results, the cases studied were divided into four groups. The first (A) consisted of seven cases of bodies stored for at least one month in refrigerated conditions, i.e., 4 °C, the second (B) consisted of indoor comparison case, (C) represented frozen corpses with visible macroscopic fungal growth, (D) represented frozen corpses without visible fungal growth, and the last data (E) showed the results from all cases.

**Fig. 1:**
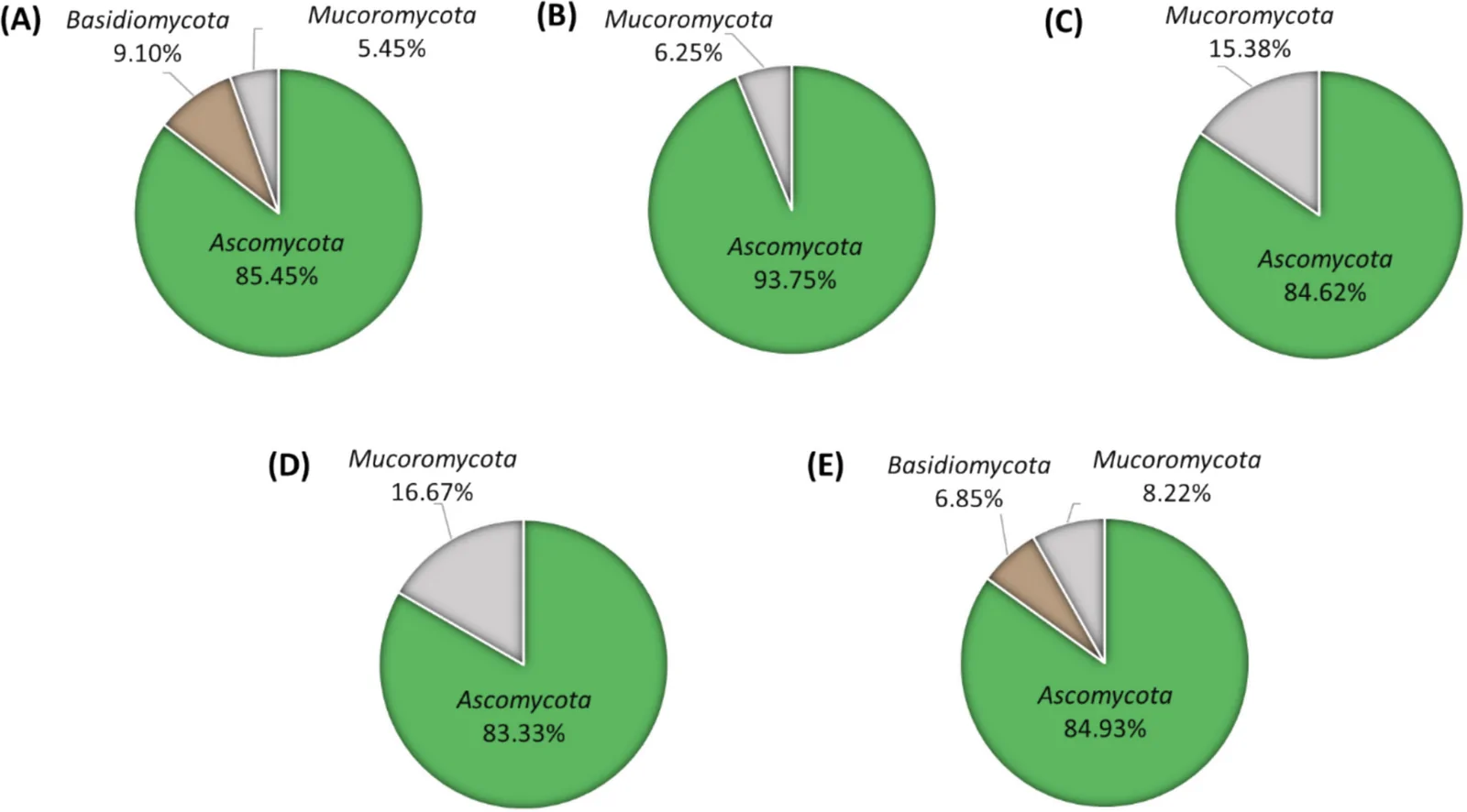
Relative frequency (%) of fungi occurrence in phylum on (**A**) corpses stored under refrigerated conditions at 4 °C, (**B**) indoor comparison case, (**C**) frozen corpses with visible macroscopic fungal growth, (**D**) frozen control corpses without visible fungal growth, and (**E**) all cases. The percentages were calculated based on the total number of fungal species identified within each group (A, n = 55 species; B, n = 16; C, n = 26; D, n = 6; E, n = 73)

The relative frequency of belonging to the phylum among the fungal species isolated in each group is shown in Fig. 1. The *Ascomycota* phylum consistently dominated each of the analyzed groups. Importantly, only on cadavers stored at 4 °C, the presence of representatives of *Basidiomycota* was noted, represented by yeast-like fungi of the genera *Apiotrichum*, *Bullera* and *Rhodotorula*. Each group contained species belonging to *Mucoromycota*, mainly represented by *Mucor* and *Linnemannia hyalina*.

**Fig. 2:**
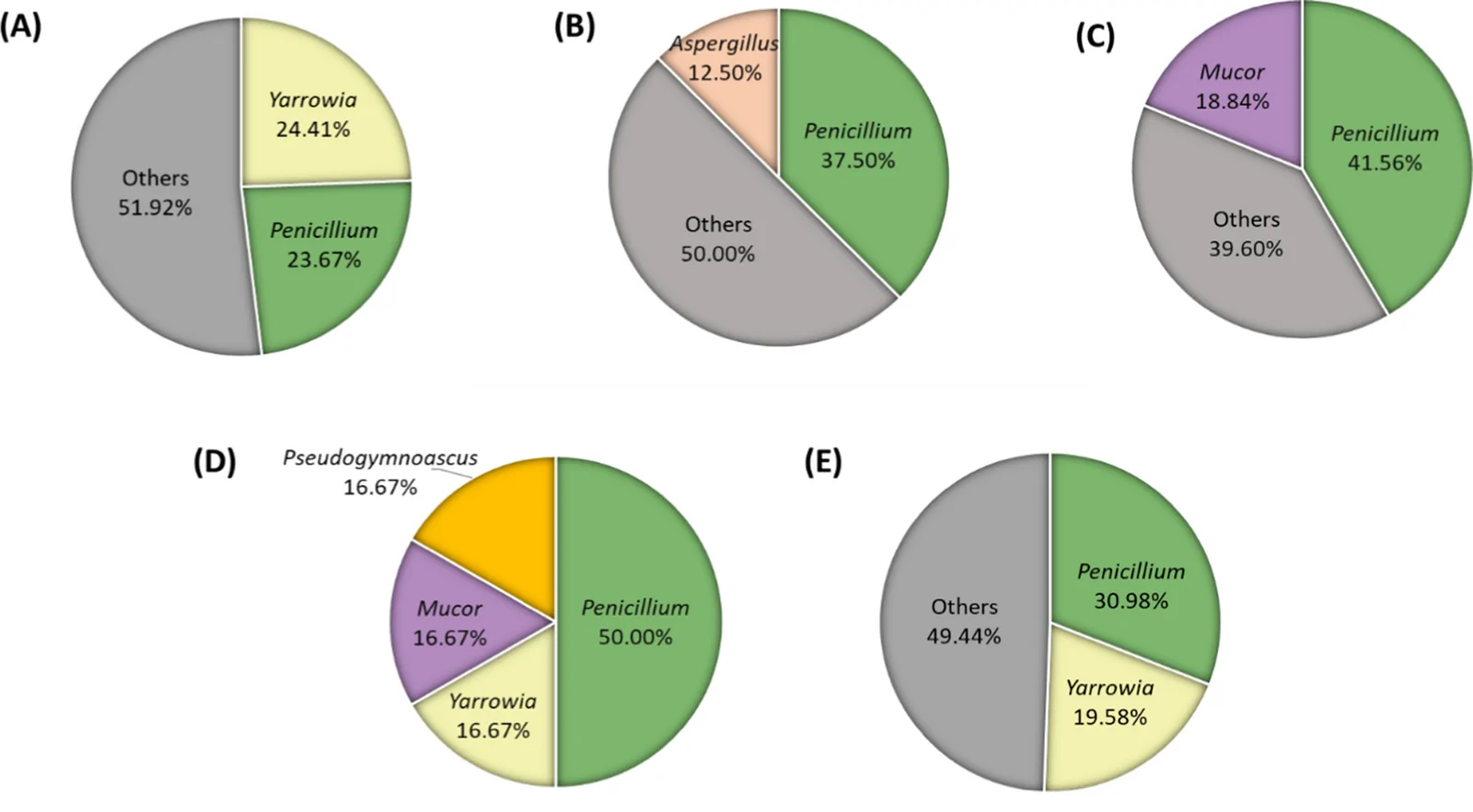
Relative frequency (%) of fungal genera from (**A**) corpses stored under refrigerated conditions at 4 °C, (**B**) indoor comparison case, (**C**) frozen cadavers with visible macroscopic fungal growth, (**D**) control frozen cadavers without visible fungal growth, and (**E**) all cases. Percentages represent the proportion of fungal species assigned to each genus within a given group. Groups A–E contained 55, 16, 26, 6, and 73 fungal species, respectively. The numbers of fungal genera identified in these groups were 32, 10, 11, 4, and 36, respectively

**Fig. 3:**
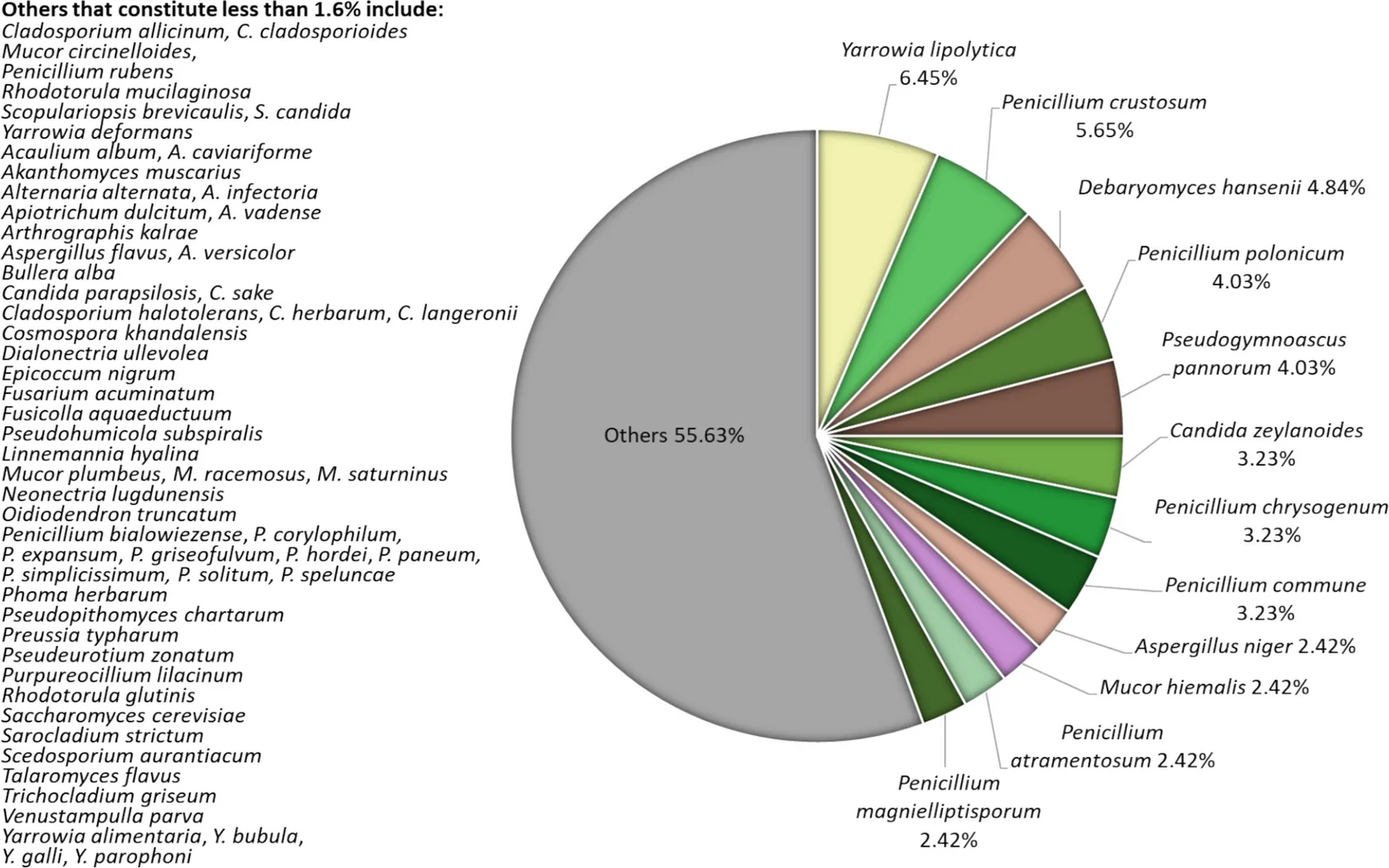
Relative frequency (%) of fungal species isolated from all cadavers included in the study. Species representing less than 1.6% of all records were grouped into the category “Others”, the composition of which is listed on the left side of the figure

Figure 2 presents the relative frequency (%) of fungal genera isolated in each group. The percentages were calculated based on the number of species representing each genus, allowing the proportional distribution and relative dominance of fungal genera among the examined cases to be illustrated. Cadavers stored at 4 °C were dominated by the genera *Penicillium* and *Yarrowia,* with *Penicillium crustosum*, *Yarrowia lipolytica*, *Debaryomyces hansenii*, and *Pseudogymnoascus pannorum* being the most frequently isolated species. In the cadaver found in apartment, the genera *Penicillium* and *Aspergillus* clearly predominated, whereas the occurrence of individual fungal species was relatively uniform. Frozen cadavers with macroscopically visible mycelium were mainly colonized by *Penicillium* and *Mucor*, with *Penicillium commune* and *Penicillium polonicum* being the most frequently isolated species. Similarly, in frozen control cadavers without macroscopically visible mycelium, *Penicillium* and *Mucor* were also common, and *Yarrowia* and *Pseudogymnoascus* were additionally detected; however, no single species clearly dominated. Overall, in all cases, the most frequently detected genera were *Penicillium* (30.98%) and *Yarrowia* (19.58%). The most frequently isolated species in all cases were *Y. lipolytica* (6.45%), *P. crustosum* (5.65%), and *D. hansenii* (4.84%). A summary of the relative contribution of each species is shown in Fig. 3.

### Culture conditions

**Fig. 4:**
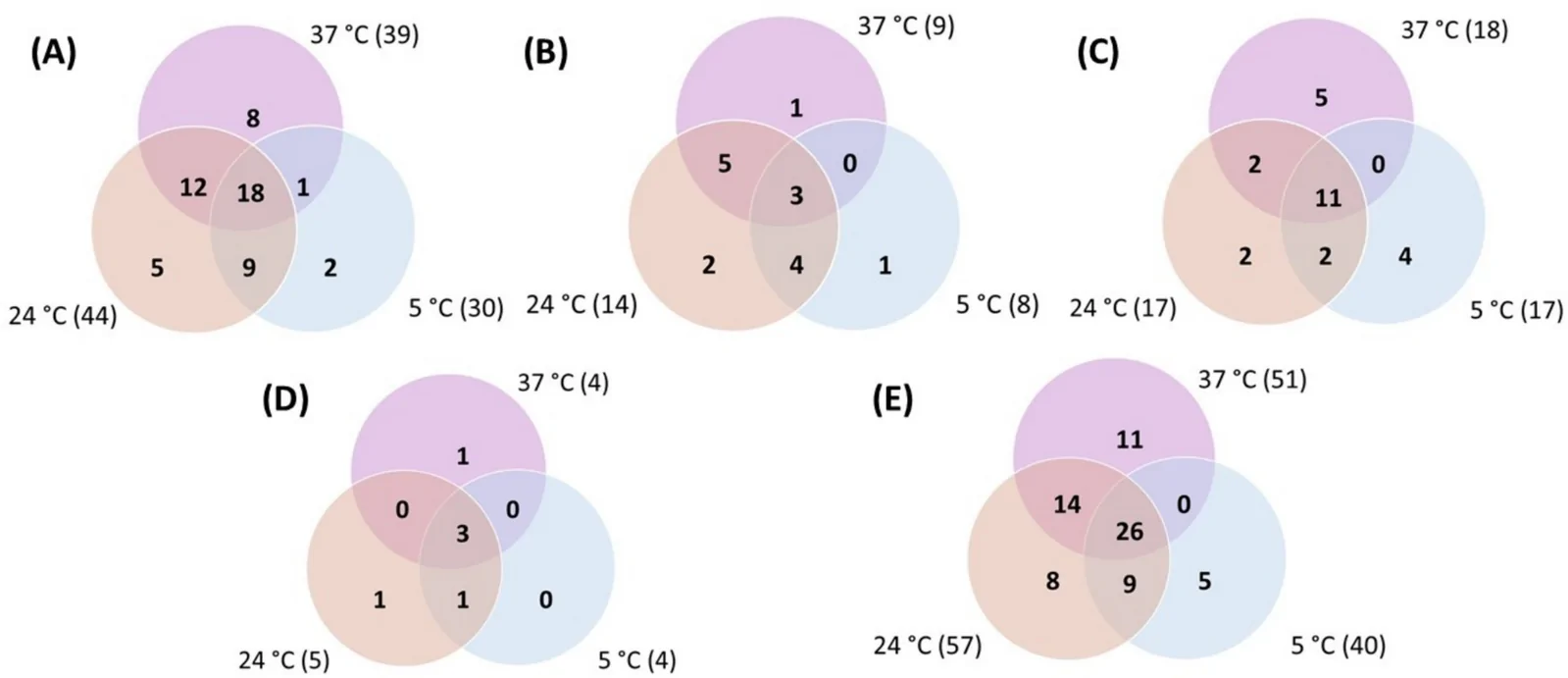
Distribution of fungal species depending on incubation temperature: (**A**) carcasses stored under refrigerated conditions at 4 °C, (**B**) indoor comparison case, (**C**) frozen cadavers with visible macroscopic fungal growth, (**D**) frozen control cadavers without visible fungal growth, and (**E**) all cases

**Fig. 5:**
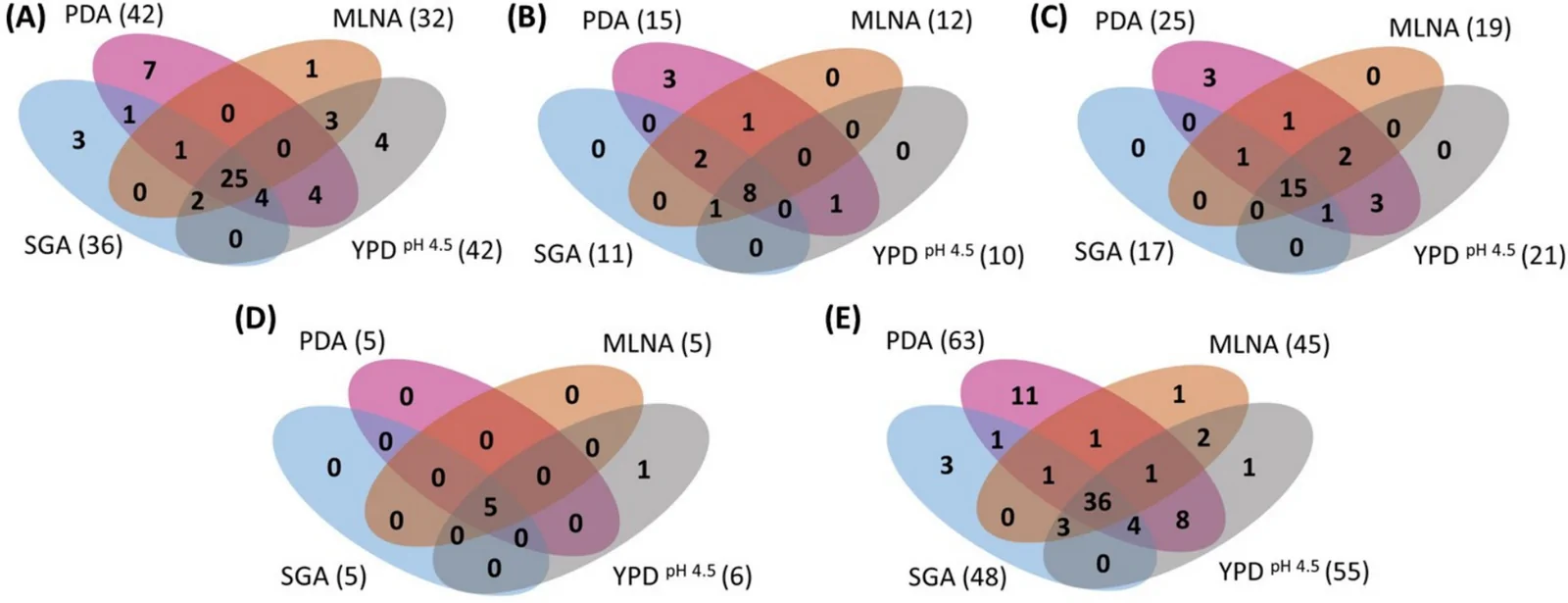
Distribution of fungal species depending on medium: (**A**) carcasses stored under refrigerated conditions at 4 °C, (**B**) indoor comparison case, (**C**) frozen cadavers with visible macroscopic fungal growth, (**D**) frozen control cadavers without visible fungal growth, and (**E**) all cases

Growth conditions, including temperature and substrate, were analyzed. The highest number of species was cultivated at 24 °C (57 species), followed by 37 °C (51 species) and 5 °C (40 species). Notably, 26 of the 73 species were able to grow at all tested temperatures (Fig. 4E). Five species appeared to be associated with lower temperatures: *Cladosporium langeronii*, *Mucor racemosus*, *Penicillium hordei*, *Penicillium speluncae*, and *Venustampulla parva* (Table S3). Among the media tested, PDA supported the highest fungal species richness and yielded the greatest number of species unique to this medium (Fig. 5). The greatest species richness was observed on PDA medium incubated at 24 °C (Table 2). Detailed information on the culture medium and incubation temperature used for each isolate is presented in Table S3, S4, S5.

**Table 2.**
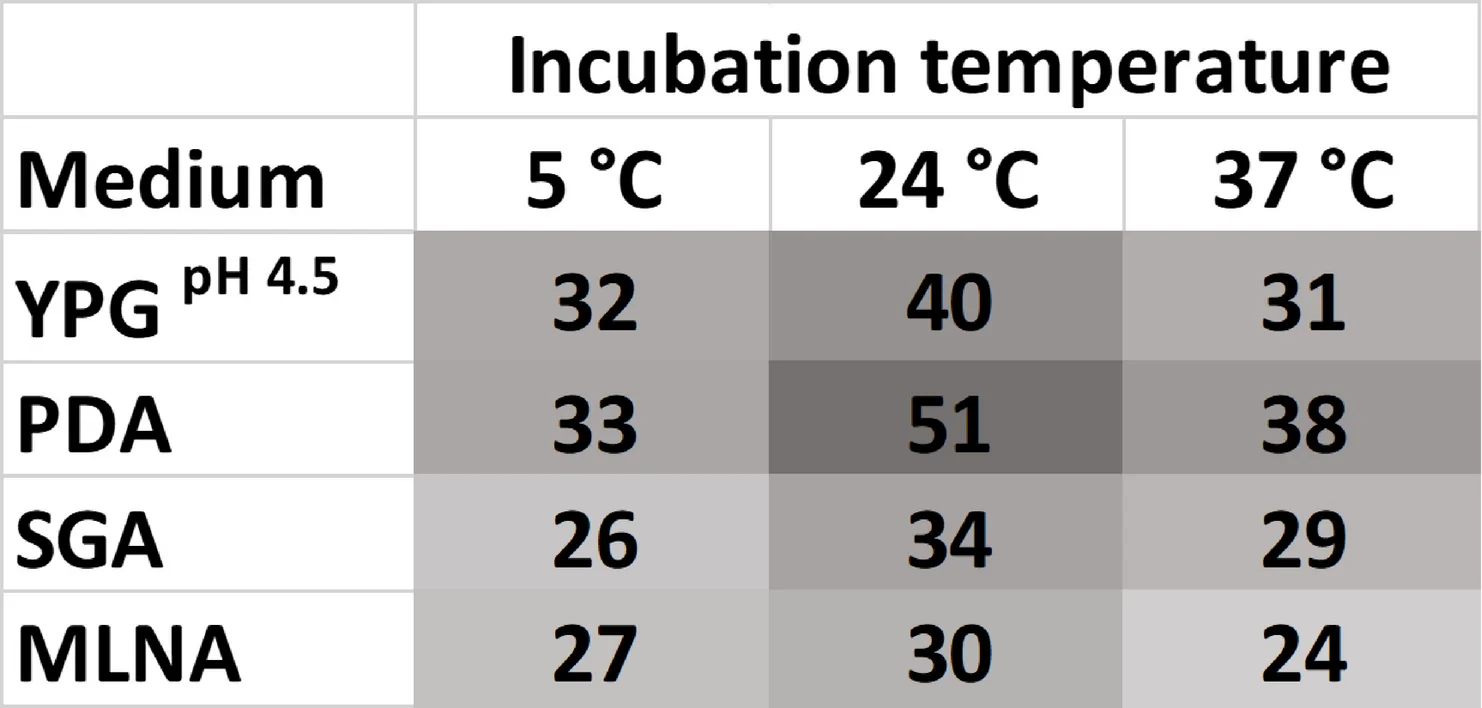
Comparison of the number of fungal species grown at different temperatures on selected medium^1^

^1^The intensity of gray shading is proportional to the number of species, with darker shades representing higher 1 species richness

### Effects of cadaver condition and cold storage duration on fungal species diversity

**Fig. 6:**
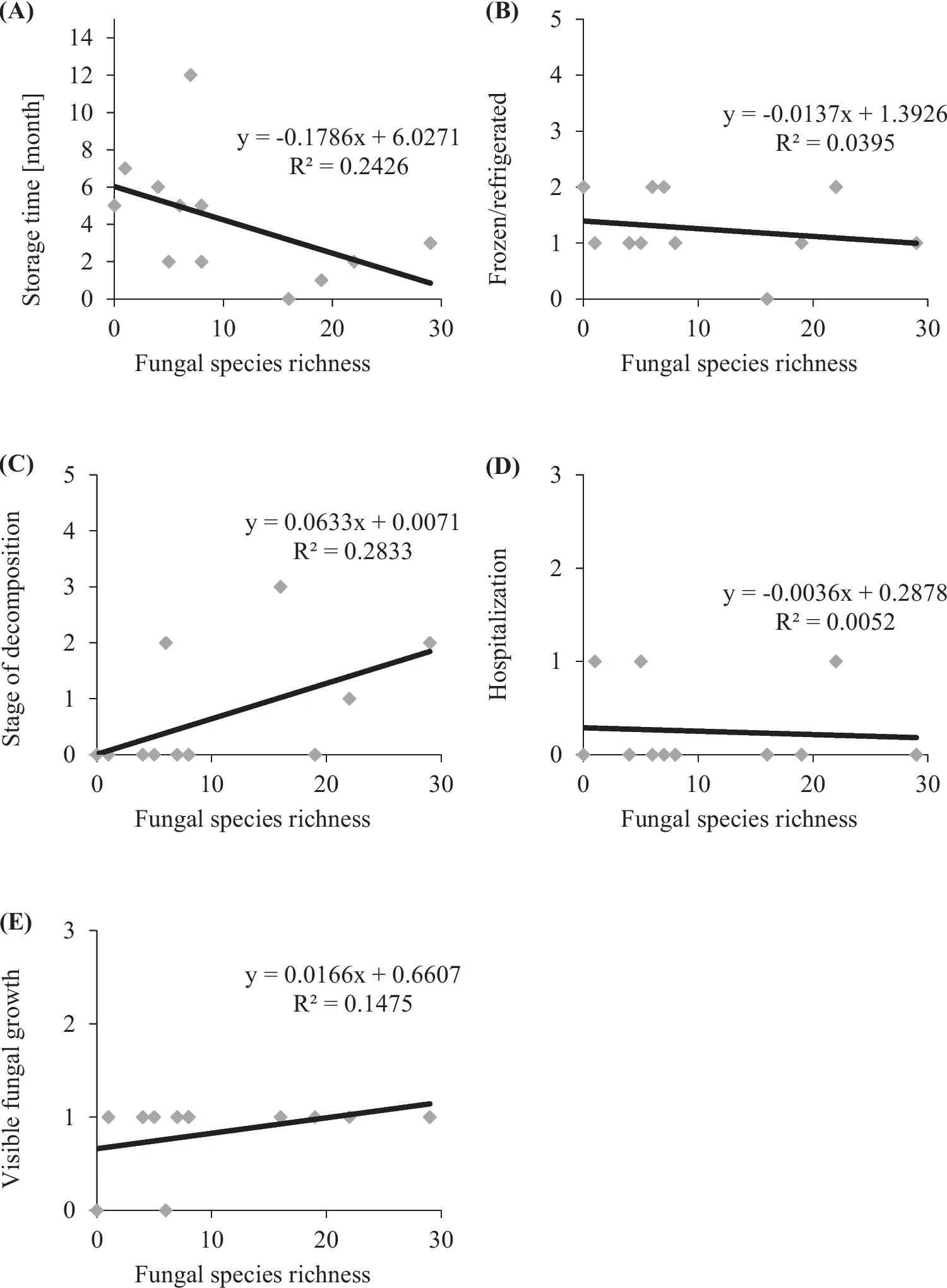
Relationship between the number of fungal species and: (**A**) storage time in a cold room (*r*_*S*_ = −0.5968, *p* = 0.0405); (**B**) freezing (*r*_*S*_ = –0.2117, *p* = 0.509); (**C**) stage of decomposition (*r*_*S*_ = 0.5264, *p* = 0.0787); (**D**) hospitalization (*r*_*S*_ = –0.1396, *p* = 0.6652); and (**E**) with macroscopically visible mycelium on body (*r*_*S*_ = 0.4542, *p* = 0.138)

Spearman's rank correlation coefficients (*r*_*S*_) were calculated to examine associations between the number of fungal species and the cadaver characteristics included in the study. Correlation analyses showed that of the five relationships examined (i.e., storage time, frozen/refrigerated, stage of cadaver decomposition, hospitalization, and visible fungal growth), one reached statistical significance (Fig. 6). A negative correlation was observed between the number of fungi species and storage time in a cold room (*r*_*S*_ = −0.5968, *p* = 0.0405).

The remaining variables did not show statistically significant correlations with fungal species richness. Nevertheless, the direction of the correlation coefficients suggested lower species richness in frozen cadavers (*r*_*S*_ = –0.2117, *p* = 0.509), and in individuals hospitalized prior to death (*r*_*S*_ = –0.1396, *p* = 0.6652), whereas higher species richness was observed in more advanced stages of decomposition (*r*_*S*_ = 0.5264, *p* = 0.0787), and those with macroscopically visible mycelium (*r*_*S*_ = 0.4542, *p* = 0.138) (Fig. 6). However, these associations should be interpreted with caution due to the lack of statistical significance.

## Discussion

Human cadavers provide a nutrient-rich substrate that supports intensive microbial activity, including the development of diverse fungal communities (Carter and Tibbett 2008; de Bruyn et al. 2025). The rate of body decomposition is influenced by multiple factors, including the cause of death, the environment in which the body is deposited, the season, scavengers, humidity, oxygen content, precipitation, and temperature (Janaway et al. 2009). Despite the widespread use of refrigerated storage for human remains, fungal communities associated with corpses under cold conditions remain poorly understood. Refrigeration alters key environmental factors governing decomposition, including temperature and moisture, yet microbial activity persists (Roberts and Dabbs 2015). Fungi, owing to their metabolic versatility and tolerance to low temperatures, may continue to colonize and transform organic substrates during storage. To the best of our knowledge, only one study has investigated fungal communities associated with human corpses stored under refrigerated conditions for an extended period, monitoring two cadavers over six weeks to characterize patterns of fungal colonization (Di Piazza et al. 2018).

The present study contributes to this field by providing a comprehensive culture-based survey of fungi associated with long-term cold storage of human remains, including both refrigerated and frozen cadavers.

Given the scarcity of available data, interpreting fungal communities associated with refrigerated cadavers remains challenging. The limited number of studies available makes it difficult to assess whether the fungal taxa recovered from refrigerated cadavers represent a characteristic component of the postmortem microbiota under cold-storage conditions or merely incidental colonizers. Establishing baseline data on fungal diversity associated with refrigerated human remains is therefore important for future forensic investigations, particularly in cases involving prolonged storage prior to autopsy or burial.

### Fungal diversity

Most isolated taxa in our study belonged to *Ascomycota*, in agreement with earlier studies on fungi associated with human remains and low-temperature environments (Gemmellaro et al. 2023; Fu et al. 2019; Paiva et al. 2023; Piepenbring et al. 2025). The dominance of the genera *Penicillium*, *Yarrowia*, and *Debaryomyces* is not surprising, as they are widely distributed on human remains across different stages of decomposition, exhibit broad hydrolytic enzyme activity, and are capable of growth across a wide temperature range (Fu et al. 2019; Sidrim et al. 2010; Spychała et al. 2025; Bolumar et al. 2008; Frisvad et al. 2004; Madzak 2021; Meena et al. 2018). Interestingly, the genus *Penicillium* was represented by 16 species, six species were identified in the genus *Yarrowia*, and the genus *Debaryomyces* was represented by *D. hansenii.* The most frequently isolated species in this study, *Y. lipolytica*, has previously been reported as one of the dominant species in the cadaver mycobiome (Fu et al. 2019). The prevalence of *P. crustosum* in this study may be associated with its ability to grow at low temperatures and its common occurrence in indoor environments and refrigerated substrates, conditions similar to those present in mortuary cold storage (Pitt and Hocking 2009; Samson et al. 2010). The halotolerant *D. hansenii* exhibits similar adaptations (Prista et al. 2005).

The vast majority of fungi isolated in our study are consistent with previous reports (Piepenbring et al. 2025). Nevertheless, numerous species were identified for the first time in association with human remains. These include: *Akanthomyces muscarius*, *Apiotrichum dulcitum*, *Apiotrichum vadense*, *Arthrographis kalrae*, *Bullera alba*, *Candida sake*, *Cladosporium halotolerans*,* C. langeronii*, *Cosmospora khandalensis*, *Oidiodendron truncatum*, *Penicillium atramentosum*, *Penicillium bialowiezense*, *Penicillium corylophilum*,* P. hordei*, *Penicillium magnielliptisporum*, *Penicillium simplicissimum*,* P. speluncae*, *Phoma herbarum*, *Pseudohumicola subspiralis*, *Pseudopithomyces chartarum*, *Preussia typharum*, *Pseudeurotium zonatum*, *Purpureocillium lilacinum*, *Scedosporium aurantiacum*, *Talaromyces flavus*, *Trichocladium griseum*, *V. parva*, *Yarrowia alimentaria*, *Yarrowia bubula*, *Yarrowia galli* and *Yarrowia parophoni*.

Although many of these species have not previously been reported from human remains, several share ecological traits that may explain their occurrence on refrigerated cadavers. These taxa include psychrotolerant fungi, saprotrophs associated with decomposing organic matter, and yeasts commonly encountered in food-related and indoor environments (Ballester-Tomás et al. 2017; Chen and Hu 2021; Vadkertiová et al. 2019). Interestingly, several species identified for the first time on human remains are commonly associated with refrigerated food products (*Yarrowia*, *Candida*, *Debaryomyces*, and *Penicillium*) (Kurtzman et al. 2011; Samson et al. 2010). Their occurrence may reflect the selective pressure imposed by prolonged storage at low temperatures and the availability of protein- and lipid-rich substrates.

The presence of such taxa also highlights the need to distinguish fungi associated with storage conditions from those acquired from the external environment prior to death, an aspect that may be relevant for the interpretation of fungal colonization in forensic investigations. These findings expand current knowledge of fungal diversity associated with human cadavers and contribute additional data to the emerging field of forensic mycology.

### Cold adaptation and temperature effects

Temperature is one of the most important factors influencing insect activity, microbial community succession, and overall decomposition dynamics. Notably, decomposition processes continue even at temperatures below 0 °C. Microorganisms remain active across a broad thermal range and persist throughout the decomposition process (Iancu et al. 2024).

Comprehensive mycological analyses require incubation at multiple temperatures and on different media to recover the widest possible diversity of fungal taxa present in a given environment. For the isolation of fungi from cold-stored corpses, we employed not only low incubation temperatures, but also temperatures optimal for mesophilic species, as well as human body temperature, in order to recover potential pathogens. The predominance of taxa recovered at 24 °C was not unexpected, as many fungal propagules are able to survive prolonged exposure to unfavorable conditions and resume growth when incubated under temperatures closer to their physiological optimum. This finding suggests that a substantial proportion of fungi associated with refrigerated cadavers may persist in a dormant or low-activity state during storage and become readily culturable when transferred to more favorable conditions.

Many of the species identified in this study are known to be adapted to cold environments and capable of growth under refrigerated conditions. It is worth noting that the genus *Penicillium*, which was the most frequently isolated in the study, was represented by several species classified as psychrotolerant/psychrophilic (*P. polonicum*, *Penicillium chrysogenum*, *Penicillium griseofulvum*) or previously isolated from environments with extremely low temperatures (*P. crustosum*, *P. commune*, *Penicillium rubens*, *Penicillium solitum*) (Cho et al. 2007; Gonçalves et al. 2013; Koleva et al. 2024; Kostadinova et al. 2017; Krumova et al. 2021; Saccomori et al. 2015; Sonjak et al. 2007). Several less frequently isolated taxa identified in this study have previously been reported from cold or cold-adapted environments, including *C. sake*, *L. hyalina*, *M. racemosus*, *O. truncatum*, *P. herbarum*, *P. pannorum*, *Rhodotorula glutinis*, and *Rhodotorula mucilaginosa*, further supporting the role of low temperature as a selective factor shaping fungal communities (Ballester-Tomás et al. 2017; Gomes et al. 2024; Hashem et al. 2023; Singh Parmar et al. 2022; Rice and Currah 2005; Telagathoti et al. 2022; Trigubovich et al. 2023; Viñarta et al. 2016). Conversely, several species (*C. langeronii, M. racemosus, P. hordei, P. speluncae* and *V. parva*) were isolated exclusively at 5 °C. *C. langeronii* belongs to a species complex comprising at least two closely related taxa, some of which have been reported from Arctic and Antarctic regions (Bensch et al. 2018). *M. racemosus* is known for its ability to grow at low temperatures and accumulate lipids (Hashem et al. 2023). *P. hordei* is characterized by growth at low temperatures and high extracellular enzyme activity (Vertot et al. 2025). *P. speluncae*, described in 2019 from a cave exhibits the ability to grow at low temperatures (Visagie et al. 2019). Although data on *V. parva* are limited, available phylogenetic analyses indicate its association with environmental isolates, including material originating from Antarctica (Untereiner et al. 2019).

### The effect of cold storage on fungal community composition

Freezing corpses is a relatively common practice used to halt or significantly slow down decomposition. In a study by Ogbanga et al. (2023) involving seven human cadavers, it was demonstrated that storage under frozen conditions preserves both the composition and abundance of the associated microbiome. However, alterations in ecological relationships within the microbial community were observed after thawing, revealing previously rare taxa (Ogbanga et al. 2023). Similarly, Gemmellaro et al. (2023) showed that different stages of decomposition exhibit distinct fungal community diversity when the remains had been initially stored at − 20 °C. On the other hand, our results show the dominance of the genus *Penicillium* on frozen corpses. Notably, some of the bodies already exhibited visible mycelial growth prior to freezing, suggesting that the initial colonization may influence the fungal communities observed during analysis. Freezing likely preserved the already established mycelium and limited further fungal growth. Consequently, the fungi detected on the frozen bodies most likely reflect the community present at the time of freezing rather than subsequent colonization or development. This observation indicates that freezing may effectively preserve the existing fungal community of the substrate rather than altering its composition. From a forensic perspective, fungi recovered from frozen cadavers may largely represent communities established before freezing rather than fungi actively growing during storage. However, freezing may also act as a selective factor, reducing the viability of some taxa while favoring cold-adapted species capable of surviving prolonged exposure to subzero temperatures. Consequently, the fungal communities recovered after thawing may not fully reflect the original composition present before freezing. The storage history of a body should therefore be considered when interpreting fungal evidence, and future studies should focus on distinguishing pre-freezing colonization from changes induced by frozen storage.

In contrast to freezing, storage at refrigeration temperatures does not completely halt microbial activity. A temperature of 4 °C allows fungi to remain metabolically active and to continue slow growth, which may result in visible fungal colonies developing on cadavers during storage. The origin of fungi on corpses may be diverse. Spores and fragments of fungal hyphae can be present on human remains from the environment (e.g., air or soil) or originate internally as part of the ante-mortem mycobiome, which may overgrow after death (Spychała et al. 2024). Once present on the substrate, these propagules may germinate and proliferate under refrigerated conditions. Many saprotrophic fungi are capable of growth at low temperatures and are considered psychrotolerant, exhibiting optimal growth at moderate temperatures but retaining the ability to proliferate under refrigerated conditions (Margesin and Miteva 2011; Robinson 2001). Consequently, storage at 4 °C may promote the development of fungi that were initially introduced from external sources or already present on the remains, leading to visible colonization during prolonged storage.

### Relationship between cadaver condition, cold storage duration, and fungal species diversity

The post-mortem microbiota depends on various factors, including the stage of decomposition, ante-mortem infections, and lifestyle (Pechal et al. 2018). In this study, we analyzed potential relationships between fungal species richness and several cadaver-related variables: storage time, storage conditions (frozen vs. refrigerated), stage of decomposition, hospitalization, and visible fungal growth. The interpretation of such relationships should be approached with caution, as fungal colonization may be influenced by numerous factors that were not assessed in the present study. Variables such as age, underlying diseases, antimicrobial therapy, immune status, lifestyle, and environmental exposure prior to death may affect both the composition and diversity of fungal communities. Consequently, observed patterns are likely the result of multiple interacting factors rather than any single variable considered in isolation. Among the analyzed factors, only storage time in a cold room showed a significant correlation with fungal diversity. The observed decrease in species richness with increasing storage duration suggests that prolonged exposure to low temperatures may act as an environmental filter, reducing the diversity of fungi capable of surviving and growing under refrigerated conditions. Such a process may favor psychrotolerant and cold-adapted taxa while limiting the persistence of less tolerant species. A similar selective effect of low temperatures has been reported in other cold environments, where temperature acts as an important ecological filter favoring psychrotolerant and cold-adapted microorganisms (Ogbanga et al. 2023; Wang et al. 2017). Although based on a limited number of cases, our findings indicate that storage history should be considered when interpreting fungal communities associated with human remains and may be relevant for future forensic investigations involving long-term refrigerated storage.

### Forensic relevance

The potential use of fungi in forensic contexts is being increasingly investigated. Their prevalence on corpses may prove highly informative; however, the development of comparable methodologies and the accumulation of more comprehensive datasets are still required.

In the context of this study, cadavers stored in morgues prior to burial may constitute a potential source of exposure to airborne fungal propagules. Interestingly, multiple species were isolated at 37 °C, including fungi of clinical significance, such as *Aspergillus flavus*, *Aspergillus niger*, and *Scopulariopsis brevicaulis*. All of these fungi are agents of opportunistic yet potentially severe infections (Iwen et al. 2012; Rudramurthy et al. 2019; Vermeulen et al. 2013). These findings, together with previously reported pathogens associated with human remains, emphasize the need for awareness of microbial hazards among personnel handling corpses (Spychała et al. 2024). This may represent an occupational risk for personnel working in morgue environments, particularly due to exposure to airborne fungal spores released during manipulation of colonized tissues. In addition to an impermeable apron and double-layer gloves, personal protective equipment should include at least N95 respirators, or full-face masks equipped with P100 filters when high concentrations of spores are anticipated, as well as protective goggles (Schwarz et al. 2015).

The large number of factors influencing post-mortem fungal diversity, combined with limited access to human cadavers, makes it difficult for scientists to draw definitive conclusions. Therefore, new data on the post-mortem fungal diversity are of great importance, and the results of our study contribute to the growing body of knowledge in this field. Future studies should aim to investigate the enzymatic capabilities of fungi isolated from human remains in order to better understand their role in the decomposition process. Investigating fungal diversity on refrigerated bodies is essential for advancing our understanding of post-mortem microbial ecology and for improving forensic interpretations of decomposition under controlled low-temperature conditions.

## Conclusions

The study characterizes microscopic fungi capable of colonizing cadavers stored in cold rooms, environments in which they can readily proliferate. A total of 73 fungal species were identified on 12 corpses, most of which were cosmopolitan. The dominant genera were *Penicillium* and *Yarrowia*, while the species *Y. lipolytica*, *P. crustosum*, *D. hansenii*, *P. polonicum*, and *P. pannorum* had the highest overall share. Analysis of the culture conditions showed that the temperature preferred by mesophilic fungi (24 °C) with PDA medium allowed the highest number of species to be isolated. Importantly, the number of fungal species decreased with the the time spent in cold storage.

In summary, refrigerated cadavers harbor a rich and complex fungal community composed of both cosmopolitan decomposers and species with known cold‑adapted traits. This study expands the current understanding of fungal diversity in forensic contexts and provides one of the most comprehensive characterizations to date of the fungal communities associated with long‑term cold storage. We encourage research into similar cases, especially given the often significant proliferation of fungi in cold storage. Moreover, this study contributes to the understanding of postmortem microbiology and environmental mycology, particularly with regard to the geographical distribution of fungi.

## Supplementary Information

Below is the link to the electronic supplementary material.

Supplementary file1 (PDF 1579 KB)

## Data Availability

All data generated or analyzed during this study are included in this article.
