# Supplementary material for: Cadavers stored under refrigerated conditions as a source of cold-adapted microscopic fungi
Source: Appl Microbiol Biotechnol. 2026 Jul 11;110:298. doi: 10.1007/s00253-026-13958-z (PMC13638924; doi:10.1007/s00253-026-13958-z)
Supplement: Supplementary file 1 [file 253_2026_13958_MOESM1_ESM.pdf]

Supplementary material

Cadavers stored under refrigerated conditions as a source of cold-adapted microscopic fungi

Applied Microbiology and Biotechnology

Klaudyna Spychała[*], Agata Piecuch, Łukasz Szleszkowski, Agata Thannhäuser, Marcin Kadej, Rafał Ogórek

*corresponding author: klaudyna.spychala@uwr.edu.pl, Department of Mycology and Genetics, Faculty of Biological Sciences, University of Wrocław, Przybyszewskiego 63, Wroclaw PL-51-148, Poland, https://orcid.org/0000-0002-3135-1386

Table S1 Anatomical locations sampled from each cadaver and the fungal species isolated from these sites[1]

Case number	Sampling sites	Identified species
I	Face – temple	*Penicillium polonicum*
	Neck	*Mucor hiemalis, Penicillium commune, P. polonicum*
	Chest	*Acaulium caviariforme, Mucor hiemalis*
	Abdomen	*Candida zeylanoides, Mucor hiemalis, M. racemosus, Penicillium commune, P. polonicum*
	Thigh	*Mucor hiemalis, Penicillium commune, P. polonicum, Yarrowia lipolytica*
II	Forearms	*Yarrowia lipolytica*
	Abdomen	*Yarrowia lipolytica*
III	Face	*Acaulium album*
	Arm	*Acaulium album, Penicillium crustosum, Penicillium solitum, Pseudogymnoascus pannorum*
	Wrist	*Pseudogymnoascus pannorum*
IV	Face	-
	Forearms	-
	Hands	-
	Chest + abdomen	-
	Thigh	-
	Shins	-
V	Face	*Pseudogymnoascus pannorum, Yarrowia lipolytica*
	Arms	*Mucor circinelloides, Pseudogymnoascus pannorum, Yarrowia lipolytica*
	Forearms	*Pseudogymnoascus pannorum, Yarrowia lipolytica*
	Hands	*Penicillium chrysogenum, P. crustosum, P.polonicum, Yarrowia lipolytica*
	Chest + abdomen	*Pseudogymnoascus pannorum, Yarrowia lipolytica*
	Thigh	*Yarrowia lipolytica*

	Shins	*Penicillium chrysogenum*, *Yarrowia lipolytica*
	Forehead	*Yarrowia deformans*, *Y. lipolytica*
	Nose	*Penicillium atramentosum*, *P. crustosum*, *Yarrowia deformans*, *Y. lipolytica*
VI	Cheeks	*Yarrowia lipolytica*
	Right hand	*Aspergillus niger*, *Debaryomyces hansenii*, *Penicillium crustosum*, *Penicillium magnielliptisporum*, *Yarrowia bubula*, *Y. deformans*, *Y. lipolytica*
	Face	*Candida zeylanoides*, *Linnemannia hyalina*, *Pseudogymnoascus pannorum*
	Neck	*Akanthomyces muscarius*, *Apiotrichum dulcitum*, *A. vadense*, *Aspergillus versicolor*, *Dialonectria ullevolea*, *Linnemannia hyalina*, *Oidiodendron truncatum*, *Pseudeurotium zonatum*, *Pseudogymnoascus pannorum*, *Sarocladium strictum*, *Trichocladium griseum*, *Yarrowia deformans*, *Y. lipolytica*, *Y. parophoni*
	Arms	*Cosmospora khandalensis*, *Pseudogymnoascus pannorum*, *Scopulariopsis candida*, *Yarrowia deformans*, *Y. lipolytica*, *Y. parophoni*
	Forearms	*Oidiodendron truncatum*, *Pseudogymnoascus pannorum*, *Yarrowia deformans*, *Y. lipolytica*, *Y. parophoni*
	Right hand	*Dialonectria ullevolea*, *Oidiodendron truncatum*, *Pseudogymnoascus pannorum*, *Scopulariopsis candida*, *Yarrowia deformans*, *Y. lipolytica*
	Left hand	*Cosmospora khandalensis*, *Mucor hiemalis*, *Oidiodendron truncatum*, *Penicillium chrysogenum*, *P. simplicissimum*, *Pseudogymnoascus pannorum*
VII	Chest	*Apiotrichum dulcitum*, *Cosmospora khandalensis*, *Fusicolla aquaeductuum*, *Mucor hiemalis*, *Oidiodendron truncatum*, *Penicillium chrysogenum*, *P. simplicissimum*, *Pseudogymnoascus pannorum*, *Scedosporium aurantiacum*, *Scopulariopsis candida*, *Trichocladium griseum*, *Yarrowia deformans*, *Y. lipolytica*, *Y. parophoni*
	Abdomen	*Cosmospora khandalensis*, *Neonectria lugdunensis*, *Penicillium simplicissimum*, *Pseudeurotium zonatum*, *Pseudogymnoascus pannorum*, *Yarrowia deformans*, *Y. lipolytica*, *Y. parophoni*
	Thighs	*Linnemannia hyalina*, *Mucor circinelloides*, *M. hiemalis*, *Oidiodendron truncatum*, *Penicillium simplicissimum*, *Pseudogymnoascus pannorum*, *Scedosporium aurantiacum*
	Shins	*Arthrographis kalrae*, *Mucor circinelloides*, *Oidiodendron truncatum*, *Penicillium simplicissimum*, *Pseudeurotium zonatum*, *Pseudohumicola subspiralis*, *Pseudogymnoascus pannorum*, *Scedosporium aurantiacum*, *Scopulariopsis brevicaulis*, *Talaromyces flavus*, *Yarrowia lipolytica*
	Dorsal surfaces of the feet	*Aspergillus versicolor*, *Debaryomyces hansenii*, *Pseudogymnoascus pannorum*, *Scedosporium aurantiacum*, *Scopulariopsis candida*, *Talaromyces flavus*, *Trichocladium griseum*, *Yarrowia deformans*, *Y. lipolytica*
	Plantar surfaces of the feet	*Mucor circinelloides*, *Pseudogymnoascus pannorum*
	Forearms	*Yarrowia alimentaria*
	Hands	*Cladosporium halotolerans*, *Penicillium corylophilum*
	Post-autopsy suture	*Cladosporium allicinum*, *Cladosporium langeronii*
VIII	Abdomen	*Penicillium bialowiezense*, *Yarrowia alimentaria*
	Thighs	*Fusarium acuminatum*, *Pseudogymnoascus pannorum*, *Yarrowia alimentaria*
	Shins	*Yarrowia alimentaria*
	Feet	*Yarrowia alimentaria*
IX	Forehead	*Debaryomyces hansenii*
	Right arm	*Debaryomyces hansenii*

	Left arm	*Aspergillus niger*, *Penicillium crustosum*
	Elbow	*Debaryomyces hansenii*, *Penicillium crustosum*
	Skin under left breast	*Debaryomyces hansenii*
	Chest	*Debaryomyces hansenii*, *Penicillium crustosum*, *Rhodotorula mucilaginosa*
	Abdomen	*Cladosporium cladosporioides*, *Penicillium crustosum*
	Post-autopsy suture	*Debaryomyces hansenii*
	Thighs	*Penicillium crustosum*
X	Nose	*Bullera alba*, *Cladosporium allicinum*, *C. cladosporioides*, *Rhodotorula mucilaginosa*, *Saccharomyces cerevisiae*
	Right nasal cavity	*Penicillium commune*, *Rhodotorula mucilaginosa*
	Left nasal cavity	*Yarrowia lipolytica*
	Right cheek	*Bullera alba*, *Penicillium crustosum*, *P. magnielliptisporum*, *P. polonicum*, *Rhodotorula mucilaginosa*
	Left cheek	*Debaryomyces hansenii*, *Epicoccum nigrum*, *Penicillium commune*, *P. crustosum*, *Purpureocillium lilacinum*, *Rhodotorula mucilaginosa*
	Oral cavity	*Candida zeylanoides*
	Abdomen	*Candida zeylanoides*, *Penicillium atramentosum*
	Male external genitalia	*Rhodotorula mucilaginosa*, *Yarrowia galli*
	Thighs	*Bullera alba*, *Rhodotorula mucilaginosa*
	Dorsal surfaces of the feet	*Candida parapsilosis*, *Phoma herbarum*
	Plantar surfaces of the feet	*Candida zeylanoides*, *Rhodotorula glutinis*
XI	Forehead	*Debaryomyces hansenii*, *Mucor saturninus*, *Penicillium atramentosum*, *P. crustosum*, *P. magnielliptisporum*, *Venustampulla parva*, *Yarrowia lipolytica*
	Eye area	*Candida sake*, *Penicillium chrysogenum*, *P. commune*, *P. magnielliptisporum*
	Cheeks	*Debaryomyces hansenii*, *Penicillium atramentosum*, *P. commune*, *P. crustosum*, *P. magnielliptisporum*
	Chin	*Debaryomyces hansenii*, *Penicillium atramentosum*, *P. chrysogenum*, *P. commune*, *P. crustosum*, *P. polonicum*, *P. rubens*, *P. solitum*, *Yarrowia lipolytica*
	Left ear	*Debaryomyces hansenii*, *Penicillium atramentosum*, *P. chrysogenum*, *P. crustosum*, *P. magnielliptisporum*, *P. solitum*
	Right ear	*Debaryomyces hansenii*, *Mucor saturninus*, *Penicillium atramentosum*, *P. chrysogenum*, *P. commune*, *P. crustosum*, *P. griseofulvum*, *P. hordei*, *P. solitum*
	Chest	*Mucor plumbeus*, *Penicillium chrysogenum*, *P. commune*, *P. crustosum*
	Left hand	*Debaryomyces hansenii*, *Mucor plumbeus*, *Penicillium atramentosum*, *P. chrysogenum*, *P. commune*, *P. crustosum*, *P. magnielliptisporum*, *P. rubens*, *P. solitum*, *Scopulariopsis candida*, *Venustampulla parva*, *Yarrowia lipolytica*
	Right hand	*Debaryomyces hansenii*, *Penicillium atramentosum*, *P. chrysogenum*, *P. commune*, *P. crustosum*, *P. magnielliptisporum*, *P. rubens*, *P. solitum*, *P. speluncae*, *Yarrowia lipolytica*
	Abdomen	*Alternaria alternata*, *Debaryomyces hansenii*, *Mucor plumbeus*, *Penicillium atramentosum*, *P. chrysogenum*, *P. crustosum*, *P. magnielliptisporum*, *P. polonicum*
	Thighs	*Debaryomyces hansenii*, *Penicillium atramentosum*, *P. chrysogenum*, *P. commune*, *P. crustosum*, *P. paneum*, *P. rubens*
	Left knee	*Debaryomyces hansenii*, *Mucor plumbeus*, *Penicillium atramentosum*, *P. commune*, *P. crustosum*, *P. polonicum*, *P. rubens*, *Pseudogymnoascus pannorum*, *Scopulariopsis candida*, *Yarrowia lipolytica*
	Right knee	*Penicillium atramentosum*, *P. commune*, *Yarrowia lipolytica*

	Shins	*Debaryomyces hansenii*, *Penicillium atramentosum*, *P. commune*, *Yarrowia lipolytica*
	Dorsal surfaces of the feet	*Debaryomyces hansenii*, *Preussia typharum*, *Yarrowia lipolytica*
	Plantar surfaces of the feet	*Yarrowia lipolytica*
XII	Chest	*Aspergillus niger*, *Candida zeylanoides*, *Penicillium rubens*, *Pitomyces chartarum*, *Yarrowia lipolytica*
	Side of the chest	*Candida zeylanoides*, *Yarrowia lipolytica*
	Abdomen	*Yarrowia lipolytica*
	Back	*Candida zeylanoides*, *Debaryomyces hansenii*, *Mucor hiemalis*, *Scopulariopsis brevicaulis*, *Yarrowia lipolytica*
	Right forearm	*Aspergillus niger*, *Mucor hiemalis*, *Penicillium chrysogenum*, *Scopulariopsis brevicaulis*, *Yarrowia lipolytica*
	Right hand	*Alternaria infectoria*, *Aspergillus flavus*, *A. niger*, *Cladosporium herbarum*, *Debaryomyces hansenii*, *Mucor hiemalis*, *Penicillium chrysogenum*, *P. commune*, *P. crustosum*, *P. rubens*, *Scopulariopsis brevicaulis*, *Yarrowia lipolytica*
	Left hand	*Aspergillus niger*, *Candida zeylanoides*, *Debaryomyces hansenii*, *Penicillium expansum*, *P. rubens*, *Yarrowia lipolytica*
	Right calf	*Aspergillus niger*, *Debaryomyces hansenii*, *Penicillium commune*, *P. rubens*, *Scopulariopsis brevicaulis*, *Yarrowia lipolytica*
	Left calf	*Aspergillus niger*, *Debaryomyces hansenii*, *Mucor hiemalis*, *Penicillium chrysogenum*, *Scopulariopsis brevicaulis*, *Yarrowia lipolytica*
	Shins	*Aspergillus niger*, *Debaryomyces hansenii*, *Penicillium chrysogenum*, *P. crustosum*, *P. polonicum*, *Scopulariopsis brevicaulis*, *Yarrowia lipolytica*
	Plantar surface of the right feet	*Aspergillus niger*, *Debaryomyces hansenii*, *Penicillium chrysogenum*, *Scopulariopsis brevicaulis*, *Yarrowia lipolytica*
	Plantar surface of the left feet	*Debaryomyces hansenii*, *Scopulariopsis brevicaulis*, *Yarrowia lipolytica*

[1]Cadavers with visible fungal growth: I, II, III, VI, VII, VII, IX, X, XI, XII. No visible mycelium on the cadavers: IV, V

Table S2 Fungi isolated from cadaver and results of BLAST (all E values were zero)

Case number	Fungi isolated from cadavers					Identity with sequence from GenBank		
	Isolate number[1, 2]	Identified species	Phylum	Family	GenBank accession no.	The sequence length (bp)	Identity, %	Accession
I	UWR_900	*Acaulium caviariforme*	*Ascomycota*	*Microascaceae*	PZ134907	508	99.80	NR_146259.1
	UWR_901	*Candida zeylanoides*	*Ascomycota*	*Debaryomycetaceae*	PZ134796	438	100	MW587743.1
	UWR_902	*Mucor hiemalis*	*Mucoromycota*	*Mucoraceae*	PZ134908	560	100	MT626048.1
	UWR_903				PZ134909	550	100	MW798364.1
	UWR_904				PZ134910	536	100	MW798364.1
	UWR_905				PZ134911	431	100	MW798364.1
	UWR_906	*Mucor racemosus*	*Mucoromycota*	*Mucoraceae*	PZ134912	369	100	OM574723.1
	UWR_907	*Penicillium commune*	*Ascomycota*	*Aspergillaceae*	PZ134913	457	100	MK660337.1
	UWR_908				PZ134914	495	100	OR085315.1
	UWR_909				PZ134915	502	100	MK267425.1
	UWR_910				PZ134916	402	100	KT315462.1
	UWR_911				PZ134917	370	100	KT315462.1
	UWR_912				PZ134918	465	100	KT315462.1
	UWR_913				PZ134919	455	100	KT315462.1
	UWR_914	*Penicillium polonicum*	*Ascomycota*	*Aspergillaceae*	PZ134920	368	100	LN827605.1
	UWR_915				PZ134921	449	100	MZ157159.1
	UWR_916				PZ134922	367	100	MT582786.1
	UWR_917				PZ134923	432	100	MZ157159.1
	UWR_918				PZ134924	385	100	LN827605.1
	UWR_919				PZ134925	488	100	MZ157159.1
	UWR_920	*Yarrowia lipolytica*	*Ascomycota*	*Dipodascaceae*	PZ134797	450	100	FM212452.1
	UWR_921				PZ134798	354	100	PV113488.1
II	UWR_922	*Yarrowia lipolytica*	*Ascomycota*	*Dipodascaceae*	PZ134799	461	100	JN112349.1
III	UWR_923	*Acaulium album*	*Ascomycota*	*Microascaceae*	PZ134926	385	100	MN991960.1

	UWR_924	*Penicillium crustosum*	*Ascomycota*	*Aspergillaceae*	PZ134927	490	100	MH427060.1
	UWR_925	*Penicillium solitum*	*Ascomycota*	*Aspergillaceae*	PZ134928	440	100	JN642222.1
	UWR_926				PZ134929	367	100	PP125168.1
	UWR_927				PZ134930	391	100	PP125168.1
	UWR_928				PZ134931	442	100	PP125168.1
	UWR_929				PZ134932	484	100	PP125168.1
	UWR_930				PZ134933	484	100	PP125168.1
	UWR_931	*Pseudogymnoascus pannorum*	*Ascomycota*	*Pseudeurotiaceae*	PZ134934	397	100	MH856298.1
	UWR_932				PZ134935	457	100	MH856298.1
	UWR_933				PZ134936	474	100	OW987424.1
	UWR_934				PZ134937	471	100	OW987424.1
	UWR_935				PZ134938	478	100	OW987424.1
	UWR_936	*Mucor circinelloides*	*Mucoromycota*	*Mucoraceae*	PZ134939	556	100	PQ654028.1
	UWR_937	*Penicillium chrysogenum*	*Ascomycota*	*Aspergillaceae*	PZ134940	495	100	MT328526.1
	UWR_938				PZ134941	494	100	MT328526.1
	UWR_939	*Penicillium crustosum*	*Ascomycota*	*Aspergillaceae*	PZ134942	497	100	MN007129.1
	UWR_940	*Penicillium polonicum*	*Ascomycota*	*Aspergillaceae*	PZ134943	438	100	MT792002.1
	UWR_941				PZ134944	486	100	OR900531.1
V	UWR_942				PZ134945	479	100	OR900531.1
	UWR_943				PZ134946	476	100	OR900531.1
	UWR_944	*Pseudogymnoascus pannorum*	*Ascomycota*	*Pseudeurotiaceae*	PZ134947	476	100	OR900531.1
	UWR_945				PZ134948	475	100	OR900531.1
	UWR_946				PZ134949	481	100	OR900531.1
	UWR_947				PZ134950	479	100	OR900531.1
	UWR_948	*Yarrowia lipolytica*	*Ascomycota*	*Dipodascaceae*	PZ134800	459	100	JX081302.1

	UWR_949				PZ134801	471	100	JX081302.1
	UWR_950				PZ134802	449	100	MG833309.1
	UWR_951				PZ134803	461	100	JX081302.1
	UWR_952				PZ134804	467	100	EF362750.1
	UWR_953				PZ134805	455	100	JX081302.1
	UWR_954				PZ134806	464	100	EF362750.1
	UWR_955				PZ134807	475	100	MN075241.1
	UWR_956				PZ134808	446	100	MG833309.1
	UWR_957				PZ134809	456	100	JX081302.1
	UWR_958				PZ134810	466	100	JX081302.1
	UWR_959				PZ134811	459	100	JX081302.1
	UWR_960				PZ134812	464	100	JX649971.1
VI	UWR_961	*Aspergillus niger*	*Ascomycota*	*Aspergillaceae*	PZ134951	414	99.52	PV657687.1
	UWR_962	*Debaryomyces hansenii*	*Ascomycota*	*Debaryomycetaceae*	PZ134813	464	99.78	MT422082.1
	UWR_963	*Penicillium atramentosum*	*Ascomycota*	*Aspergillaceae*	PZ134952	474	100	PV109845.1
	UWR_964	*Penicillium crustosum*	*Ascomycota*	*Aspergillaceae*	PZ134953	372	100	ON127404.1
	UWR_965				PZ134954	370	100	ON127404.1
	UWR_966	*Penicillium magnielliptisporum*	*Ascomycota*	*Aspergillaceae*	PZ134955	356	100	PP578751.1
	UWR_967	*Yarrowia bubula*	*Ascomycota*	*Dipodascaceae*	PZ134814	381	100	NG_059943.1
	UWR_968	*Yarrowia deformans*	*Ascomycota*	*Dipodascaceae*	PZ134815	468	100	MK394171.1
	UWR_969				PZ134816	457	100	MK394171.1
	UWR_970				PZ134817	469	100	MK394171.1
	UWR_971	*Yarrowia lipolytica*	*Ascomycota*	*Dipodascaceae*	PZ134818	489	99.80	OR536331.1
VII	UWR_972	*Akanthomyces muscarius*	*Ascomycota*	*Cordycipitaceae*	PZ134956	520	100	OQ073902.1
	UWR_973	*Apiotrichum dulcitum*	*Basidiomycota*	*Trichosporonaceae*	PZ134819	505	99.60	PV199145.1
	UWR_974	*Apiotrichum vadense*	*Basidiomycota*	*Trichosporonaceae*	PZ134820	511	100	JN939477.1
	UWR_975	*Arthrographis kalrae*	*Ascomycota*	*Eremomycetaceae*	PZ134821	511	99.80	MN794346.1

UWR_976	*Aspergillus versicolor*	*Ascomycota*	*Aspergillaceae*	PZ134957	492	100	KF815587.1
UWR_977	*Candida zeylanoides*	*Ascomycota*	*Debaryomycetaceae*	PZ134822	381	99.74	KY512442.1
UWR_978	*Cosmospora khandalensis*	*Ascomycota*	*Nectriaceae*	PZ134958	487	99.79	OL703550.1
UWR_979	*Debaryomyces hansenii*	*Ascomycota*	*Debaryomycetaceae*	PZ134823	467	100	GQ352526.1
UWR_980	*Dialonectria ullevolea*	*Ascomycota*	*Nectriaceae*	PZ134824	527	100	MH866681.1
UWR_981				PZ134825	487	100	MH866681.1
UWR_982	*Fusicolla aquaeductuum*	*Ascomycota*	*Nectriaceae*	PZ134826	530	100	MH087221.1
UWR_983	*Linnemannia hyalina*	*Mucoromycota*	*Mortierellaceae*	PZ134959	552	99.82	PQ113889.1
UWR_984	*Mucor circinelloides*	*Mucoromycota*	*Mucoraceae*	PZ134960	578	99.13	MW007765.1
UWR_985				PZ134961	573	99.83	MW007765.1
UWR_986	*Mucor hiemalis*	*Mucoromycota*	*Mucoraceae*	PZ134962	583	100	DQ118992.1
UWR_987	*Neonectria lugdunensis*	*Ascomycota*	*Nectriaceae*	PZ134963	467	100	OK338524.1
UWR_988	*Oidiodendron truncatum*	*Ascomycota*	*Myxotrichaceae*	PZ134964	469	100	NR_111036.1
UWR_989				PZ134965	489	100	LC317709.1
UWR_990				PZ134966	454	100	JN104533.1
UWR_991				PZ134967	490	100	LC317709.1
UWR_992	*Penicillium chrysogenum*	*Ascomycota*	*Aspergillaceae*	PZ134968	509	100	PQ329214.1
UWR_993				PZ134969	384	100	MG269835.1
UWR_994	*Penicillium simplicissimum*	*Ascomycota*	*Aspergillaceae*	PZ134970	523	100	OP179089.1
UWR_995				PZ134971	507	100	OP179089.1
UWR_996				PZ134972	498	100	OP179089.1
UWR_997	*Pseudeurotium zonatum*	*Ascomycota*	*Pseudeurotiaceae*	PZ134973	471	99.79	MH864346.1
UWR_998	*Pseudohumicola subspiralis*	*Ascomycota*	*Chaetomiaceae*	PZ134974	473	100	MH861822.1
UWR_999	*Pseudogymnoascus pannorum*	*Ascomycota*	*Pseudeurotiaceae*	PZ134975	430	100	MH854615.1
UWR_1000				PZ134976	484	100	MZ447498.1

	UWR_1001				PZ134977	482	100	MZ447498.1
	UWR_1002				PZ134978	485	100	OR900531.1
	UWR_1003				PZ134979	377	100	MZ447498.1
	UWR_1004				PZ134980	490	100	OL334746.1
	UWR_1005				PZ134981	481	100	MN879385.1
	UWR_1006				PZ134982	487	100	OR900531.1
	UWR_1007				PZ134983	370	100	MW018710.1
	UWR_1008				PZ134984	407	100	MH856116.1
	UWR_1009	*Sarocladium strictum*	*Ascomycota*	*Sarocladiaceae*	PZ134827	410	100	OK337776.1
	UWR_1010	*Scedosporium aurantiacum*	*Ascomycota*	*Microascaceae*	PZ134985	546	100	OQ625630.1
	UWR_1011				PZ134986	550	100	OQ625630.1
	UWR_1012				PZ134987	410	100	OQ625630.1
	UWR_1013	*Scopulariopsis brevicaulis*	*Ascomycota*	*Microascaceae*	PZ134988	566	100	OW987504.1
	UWR_1014	*Scopulariopsis candida*	*Ascomycota*	*Microascaceae*	PZ134989	429	99.77	KX924015.1
	UWR_1015	*Talaromyces flavus*	*Ascomycota*	*Trichocomaceae*	PZ134990	493	100	MT074666.1
	UWR_1016				PZ134991	489	100	MT074667.1
	UWR_1017	*Trichocladium griseum*	*Ascomycota*	*Chaetomiaceae*	PZ134992	497	100	AB625590.1
	UWR_1018				PZ134993	473	100	AB625590.1
	UWR_1019	*Yarrowia deformans*	*Ascomycota*	*Dipodascaceae*	PZ134828	366	100	MW049213.1
	UWR_1020				PZ134829	457	100	MW049213.1
	UWR_1021				PZ134830	413	100	MW049213.1
	UWR_1022	*Yarrowia lipolytica*	*Ascomycota*	*Dipodascaceae*	PZ134831	377	100	OR536331.1
	UWR_1023				PZ134832	451	100	OP136087.1
	UWR_1024				PZ134833	423	99.76	EU327096.1
	UWR_1025				PZ134834	360	100	EU327096.1
	UWR_1026	*Yarrowia parophoni*	*Ascomycota*	*Dipodascaceae*	PZ134835	448	100	KY457256.1
	UWR_1027				PZ134836	463	99.78	KY457256.1
VIII	UWR_1028	*Cladosporium allicinum*	*Ascomycota*	*Cladosporiaceae*	PZ134994	463	100	OW983749.1
	UWR_1029	*Cladosporium halotolerans*	*Ascomycota*	*Cladosporiaceae*	PZ134995	463	100	OR978457.1

	UWR_1030	*Cladosporium langeronii*	*Ascomycota*	*Cladosporiaceae*	PZ134996	455	100	KP701931.1
	UWR_1031	*Fusarium acuminatum*	*Ascomycota*	*Nectriaceae*	PZ134997	489	100	PP708927.1
	UWR_1032	*Penicillium bialowiezense*	*Ascomycota*	*Aspergillaceae*	PZ134998	502	100	OU989429.1
	UWR_1033	*Penicillium corylophilum*	*Ascomycota*	*Aspergillaceae*	PZ134999	370	100	MF475908.1
	UWR_1034	*Pseudogymnoascus pannorum*	*Ascomycota*	*Pseudeurotiaceae*	PZ135000	487	100	OP889572.1
	UWR_1035	*Yarrowia alimentaria*	*Ascomycota*	*Dipodascaceae*	PZ134837	472	100	NG_058277.1
	UWR_1036				PZ134838	460	100	NG_058277.1
	UWR_1037				PZ134839	456	100	NG_058277.1
	UWR_1038				PZ134840	465	100	NG_058277.1
	UWR_1039				PZ134841	472	100	NG_058277.1
	UWR_1040				PZ134842	477	100	NG_058277.1
	UWR_1041				PZ134843	351	100	NG_058277.1
	UWR_1042	*Aspergillus niger*	*Ascomycota*	*Aspergillaceae*	PZ135001	417	100	KY465752.1
	UWR_1043	*Cladosporium cladosporioides*	*Ascomycota*	*Cladosporiaceae*	PZ135002	450	100	OQ211150.1
	UWR_1044				PZ135003	417	100	PV801988.1
IX	UWR_1045	*Debaryomyces hansenii*	*Ascomycota*	*Debaryomycetaceae*	PZ134844	474	100	MT422082.1
	UWR_1046				PZ134845	474	100	MT422082.1
	UWR_1047				PZ134846	509	100	MT422082.1
	UWR_1048				PZ134847	474	100	MT422082.1
	UWR_1049				PZ134848	520	100	KY512409.1
	UWR_1050				PZ134849	498	100	MT422082.1
	UWR_1051				PZ134850	481	100	MT180741.1
	UWR_1052	*Penicillium crustosum*	*Ascomycota*	*Aspergillaceae*	PZ135004	495	100	KF572147.1
	UWR_1053				PZ135005	417	100	MK671621.1
	UWR_1054				PZ135006	472	100	PQ069635.1
	UWR_1055				PZ135007	496	100	PQ069635.1
	UWR_1056	*Rhodotorula mucilaginosa*	*Basidiomycota*	*Sporidiobolaceae*	PZ134851	503	100	MT550663.1
X	UWR_1057	*Bullera alba*	*Basidiomycota*	*Bulleraceae*	PZ134852	506	99.60	MG009570.1

UWR_1058				PZ134853	368	99.73	MG009570.1
UWR_1059	*Cladosporium allicinum*	*Ascomycota*	*Cladosporiaceae*	PZ135008	368	99.73	MG009570.1
UWR_1060	*Cladosporium cladosporioides*	*Ascomycota*	*Cladosporiaceae*	PZ135009	367	100	MZ159857.1
UWR_1061	*Candida parapsilosis*	*Ascomycota*	*Debaryomycetaceae*	PZ134854	371	99.46	JQ965866.1
UWR_1062	*Candida zeylanoides*	*Ascomycota*	*Debaryomycetaceae*	PZ134855	380	99.74	PV113482.1
UWR_1063				PZ134856	362	99.72	MT422087.1
UWR_1064				PZ134857	436	99.54	MT422087.1
UWR_1065	*Debaryomyces hansenii*	*Ascomycota*	*Debaryomycetaceae*	PZ134858	498	100	MN704642.1
UWR_1066	*Epicoccum nigrum*	*Ascomycota*	*Didymellaceae*	PZ135010	381	100	MT573480.1
UWR_1067				PZ135011	457	100	OL691149.1
UWR_1068	*Penicillium atramentosum*	*Ascomycota*	*Aspergillaceae*	PZ135012	431	100	PV109845.1
UWR_1069	*Penicillium commune*	*Ascomycota*	*Aspergillaceae*	PZ135013	516	100	KM115157.1
UWR_1070				PZ135014	355	100	KM115157.1
UWR_1071				PZ135015	377	100	MK660351.1
UWR_1072				PZ135016	380	99.21	MK660351.1
UWR_1073	*Penicillium crustosum*	*Ascomycota*	*Aspergillaceae*	PZ135017	419	100	KF938470.1
UWR_1074				PZ135018	473	100	OR665155.1
UWR_1075	*Penicillium magnielliptisporum*	*Ascomycota*	*Aspergillaceae*	PZ135019	501	100	OP179044.1
UWR_1076				PZ135020	445	100	OP179044.1
UWR_1077	*Penicillium polonicum*	*Ascomycota*	*Aspergillaceae*	PZ135021	460	100	MT529240.1
UWR_1078	*Phoma herbarum*	*Ascomycota*	*Didymellaceae*	PZ135022	450	100	OQ376721.1
UWR_1079	*Purpureocillium lilacinum*	*Ascomycota*	*Ophiocordycipitaceae*	PZ135023	400	100	MT530278.1
UWR_1080	*Rhodotorula glutinis*	*Basidiomycota*	*Sporidiobolaceae*	PZ134859	538	100	MN700872.1
UWR_1081	*Rhodotorula mucilaginosa*	*Basidiomycota*	*Sporidiobolaceae*	PZ134860	473	100	KY109063.1

XI	UWR_1082	*Saccharomyces cerevisiae*	*Ascomycota*	*Saccharomycetaceae*	PZ134861	368	99.73	MN464141.1
	UWR_1083	*Yarrowia galli*	*Ascomycota*	*Dipodascaceae*	PZ134862	412	99.76	KY106459.1
	UWR_1084	*Yarrowia lipolytica*	*Ascomycota*	*Dipodascaceae*	PZ134863	373	99.73	PV113484.1
	UWR_1085				PZ134864	400	99.50	PV113493.1
	UWR_1086	*Alternaria alternata*	*Ascomycota*	*Pleosporaceae*	PZ135024	444	100	MW723999.1
	UWR_1087	*Candida sake*	*Ascomycota*	*Debaryomycetaceae*	PZ134865	498	99.79	MT577809.1
	UWR_1088	*Debaryomyces hansenii*	*Ascomycota*	*Debaryomycetaceae*	PZ134866	523	100	MT422082.1
	UWR_1089				PZ134867	530	100	MT422082.1
	UWR_1090				PZ134868	463	100	MT422082.1
	UWR_1091				PZ134869	528	100	MT422082.1
	UWR_1092				PZ134870	509	100	MT422082.1
	UWR_1093				PZ134871	507	100	MT422082.1
	UWR_1094				PZ134872	532	100	OR649298.1
	UWR_1095	*Mucor plumbeus*	*Mucoromycota*	*Mucoraceae*	PZ135025	550	100	OW984273.1
	UWR_1096				PZ135026	557	100	OW984273.1
	UWR_1097	*Mucor saturninus*	*Mucoromycota*	*Mucoraceae*	PZ135027	372	100	JN206074.1
	UWR_1098	*Penicillium atramentosum*	*Ascomycota*	*Aspergillaceae*	PZ135028	404	100	OP178990.1
	UWR_1099				PZ135029	425	100	OP178990.1
	UWR_1100				PZ135030	397	100	HQ115681.1
	UWR_1101				PZ135031	456	100	OP178990.1
	UWR_1102				PZ135032	507	100	OP178990.1
	UWR_1103				PZ135033	448	100	OP178990.1
	UWR_1104				PZ135034	355	100	HQ115681.1
	UWR_1105				PZ135035	509	100	OP178990.1
	UWR_1106	*Penicillium chrysogenum*	*Ascomycota*	*Aspergillaceae*	PZ135036	461	100	MN839773.1
	UWR_1107				PZ135037	472	100	MN839773.1
	UWR_1108				PZ135038	487	100	MN839773.1
	UWR_1109	*Penicillium commune*	*Ascomycota*	*Aspergillaceae*	PZ135039	500	100	MK660351.1
	UWR_1110				PZ135040	492	100	PX308024.1
	UWR_1111				PZ135041	447	100	PX308024.1
	UWR_1112				PZ135042	427	100	MH481304.1

UWR_1113	*Penicillium crustosum*	*Ascomycota*	*Aspergillaceae*	PZ135043	531	100	KC329623.1
UWR_1114				PZ135044	406	100	KT315462.1
UWR_1115				PZ135045	489	100	OP237287.1
UWR_1116				PZ135046	374	100	KF938470.1
UWR_1117				PZ135047	402	100	KF938470.1
UWR_1118				PZ135048	500	100	OR673669.1
UWR_1119				PZ135049	392	100	KF938470.1
UWR_1120				PZ135050	444	100	MH427060.1
UWR_1121				PZ135051	434	100	MH427060.1
UWR_1122				PZ135052	443	100	MH427060.1
UWR_1123	*Penicillium griseofulvum*	*Ascomycota*	*Aspergillaceae*	PZ135053	494	100	DQ339549.1
UWR_1124	*Penicillium hordei*	*Ascomycota*	*Aspergillaceae*	PZ135054	490	100	MK226540.1
UWR_1125	*Penicillium magnielliptisporum*	*Ascomycota*	*Aspergillaceae*	PZ135055	438	100	OP179044.1
UWR_1126				PZ135056	472	100	OP179044.1
UWR_1127				PZ135057	404	100	OP179044.1
UWR_1128				PZ135058	412	100	OP179044.1
UWR_1129				PZ135059	367	100	OP179044.1
UWR_1130				PZ135060	416	100	OP179044.1
UWR_1131				PZ135061	358	100	PP578751.1
UWR_1132				PZ135062	492	100	OP179044.1
UWR_1133	*Penicillium paneum*	*Ascomycota*	*Aspergillaceae*	PZ135063	484	100	KX664403.1
UWR_1134	*Penicillium polonicum*	*Ascomycota*	*Aspergillaceae*	PZ135064	368	100	KU321540.1
UWR_1135	*Penicillium rubens*	*Ascomycota*	*Aspergillaceae*	PZ135065	389	100	KP641164.1
UWR_1136	*Penicillium solitum*	*Ascomycota*	*Aspergillaceae*	PZ135066	415	100	JN642222.1
UWR_1137				PZ135067	431	100	JN642222.1
UWR_1138	*Penicillium speluncae*	*Ascomycota*	*Aspergillaceae*	PZ135068	491	100	MG490864.1
UWR_1139	*Preussia typharum*	*Ascomycota*	*Sporormiaceae*	PZ135069	435	100	MT072060.1
UWR_1140	*Pseudogymnoascus pannorum*	*Ascomycota*	*Pseudeurotiaceae*	PZ135070	487	100	OR900531.1

	UWR_1141	*Scopulariopsis candida*	*Ascomycota*	*Microascaceae*	PZ135071	364	99.72	KX924012.1
	UWR_1142	*Venustampulla parva*	*Ascomycota*	*Pleuroascaceae*	PZ135072	394	100	MT732898.1
	UWR_1143	*Yarrowia lipolytica*	*Ascomycota*	*Dipodascaceae*	PZ134873	470	100	MH545931.1
	UWR_1144				PZ134874	485	100	KY110188.1
	UWR_1145				PZ134875	489	100	FJ438480.1
	UWR_1146	*Alternaria infectoria*	*Ascomycota*	*Pleosporaceae*	PZ135073	407	100	MT561399.1
	UWR_1147	*Aspergillus flavus*	*Ascomycota*	*Aspergillaceae*	PZ135074	442	100	OM617906.1
	UWR_1148	*Aspergillus niger*	*Ascomycota*	*Aspergillaceae*	PZ135075	499	100	MF152935.1
	UWR_1149	*Candida zeylanoides*	*Ascomycota*	*Debaryomycetaceae*	PZ134876	519	99.61	MW587741.1
	UWR_1150	*Cladosporium herbarum*	*Ascomycota*	*Cladosporiaceae*	PZ135076	472	100	ON712476.1
	UWR_1151	*Debaryomyces hansenii*	*Ascomycota*	*Debaryomycetaceae*	PZ134877	375	99.20	KY511887.1
	UWR_1152				PZ134878	401	99.25	KY511954.1
	UWR_1153				PZ134879	396	99.24	KY511954.1
	UWR_1154	*Mucor hiemalis*	*Mucoromycota*	*Mucoraceae*	PZ135077	481	100	MT366055.1
XII	UWR_1155	*Penicillium chrysogenum*	*Ascomycota*	*Aspergillaceae*	PZ135078	511	100	PP053032.1
	UWR_1156				PZ135079	507	100	PP053032.1
	UWR_1157	*Penicillium commune*	*Ascomycota*	*Aspergillaceae*	PZ135080	508	100	MK660354.1
	UWR_1158	*Penicillium crustosum*	*Ascomycota*	*Aspergillaceae*	PZ135081	425	100	MT872085.1
	UWR_1159				PZ135082	381	100	KF938470.1
	UWR_1160				PZ135083	399	100	KF938470.1
	UWR_1161	*Penicillium expansum*	*Ascomycota*	*Aspergillaceae*	PZ135084	361	100	MK660357.1
	UWR_1162	*Penicillium polonicum*	*Ascomycota*	*Aspergillaceae*	PZ135085	495	100	MT529889.1
	UWR_1163	*Penicillium rubens*	*Ascomycota*	*Aspergillaceae*	PZ135086	424	100	KP641164.1
	UWR_1164	*Pseudopithomyces chartarum*	*Ascomycota*	*Didymosphaeriaceae*	PZ135087	462	100	OR691764.1

UWR_1165	*Scopulariopsis brevicaulis*	*Ascomycota*	*Microascaceae*	PZ135088	383	100	MH459001.1
UWR_1166	*Yarrowia lipolytica*	*Ascomycota*	*Dipodascaceae*	PZ134880	375	99.73	MH545931.1
UWR_1167				PZ134881	422	99.76	PV113496.1
UWR_1168				PZ134882	440	99.77	MH545931.1
UWR_1169				PZ134883	419	100	MK394170.1

[1] Using the ITS1 and ITS4 primer pairs, the following sequences were obtained: UWR_900, UWR_902 - UWR_919, UWR_923 - UWR_947, UWR_961, UWR_963 - UWR_966, UWR_972, UWR_976, UWR_978, UWR_983 - UWR_1008, UWR_1010 - UWR_1018, UWR_1028 - UWR_1034, UWR_1042 - UWR_1044, UWR_1052 - UWR_1055, UWR_1059, UWR_1060, UWR_1066 - UWR_1079, UWR_1086, UWR_1095 - UWR_1142, UWR_1146 - UWR_1148, UWR_1150, UWR_1154 - UWR_1165.

[2] The NL-1 and NL-4 starter pair was used for: UWR_901, UWR_920 - UWR_922, UWR_948 - UWR_960, UWR_962, UWR_967 - UWR_971, UWR_973 - UWR_975, UWR_977, UWR_979 - UWR_982, UWR_1009, UWR_1019 - UWR_1027, UWR_1035 - UWR_1041, UWR_1045 - UWR_1051, UWR_1056 - UWR_1058, UWR_1061 - UWR_1065, UWR_1080 - UWR_1085, UWR_1087 - UWR_1094, UWR_1143 - UWR_1145, UWR_1149, UWR_1151 – UWR, 1153, UWR_1166 - UWR_1169.

Table S3 Fungal isolates identified in this study at 5 °C relative to the culture medium. a – YPD $^{pH\ 4.5}$, b – PDA, c – SGA, d – MLNA

Legend: **YPG $^{pH\ 4.5}$** (YPG medium reduced to pH 4.5 and containing chloramphenicol); **PDA** (Potato Dextrose Agar); **SGA** (Sabouraud's Glucose Agar); **MLNA** (Modified Leeming–Notman agar)

**Fungi species**	**Isolate no.**	**Case number**											
		**I**	**II**	**III**	**IV**	**V**	**VI**	**VII**	**VIII**	**IX**	**X**	**XI**	**XII**
Acaulium album	UWR_923			+ [a, b, c, d]									
Acaulium caviariforme	UWR_900	+ [a, b, d]											
Akanthomyces muscarius	UWR_972												
Alternaria alternata	UWR_1086												
Alternaria infectoria	UWR_1146												

Apiotrichum dulcitum	UWR_973	+[a]				
Apiotrichum vadense	UWR_974					
Arthrographis kalrae	UWR_975					
Aspergillus flavus	UWR_1147					
Aspergillus niger	UWR_961					
	UWR_1042					
	UWR_1148					
Aspergillus versicolor	UWR_976	+[c, d]				
Bullera alba	UWR_1057				+[b]	
	UWR_1058				+[a]	
Candida parapsilosis	UWR_1061					
Candida sake	UWR_1087					
Candida zeylanoides	UWR_901					
	UWR_1062				+[a]	
	UWR_1063					
	UWR_1064					
	UWR_977					
	UWR_1149					+[a, b, c, d]
Cladosporium allicinum	UWR_1028		+[b, d]			
	UWR_1059					
Cladosporium cladosporioides	UWR_1043			+[a, b, c, d]		
	UWR_1044					
	UWR_1060					
Cladosporium halotolerans	UWR_1029					
Cladosporium herbarum	UWR_1150					
Cladosporium langeronii	UWR_1030					
Cosmospora khandalensis	UWR_978					
Debaryomyces hansenii	UWR_962					
	UWR_1045			+[b, c, d]		
	UWR_1046			-		
	UWR_1047			+[b, c, d]		
	UWR_1048			+[a, b, c]		
	UWR_1049			-		

	UWR_1050				+[a, b]			
	UWR_1051				+[b, c, d]			
	UWR_1065							
	UWR_1088						+[a, b, c, d]	
	UWR_1089						+[a, b, c, d]	
	UWR_1090						+[a, b, c, d]	
	UWR_1091						+[a, b, c, d]	
	UWR_1092						+[a, b, c, d]	
	UWR_1093						+[a, b, c, d]	
	UWR_1094						+[a, b, c, d]	
	UWR_979							
	UWR_1151							+[a, b, c, d]
	UWR_1152							
	UWR_1153							+[a, b, c, d]
Dialonectria ullevolea	UWR_980							
	UWR_981							
Epicoccum nigrum	UWR_1066							
	UWR_1067					+[a, c]		
Fusarium acuminatum	UWR_1031							
Fusicolla aquaeductuum	UWR_982							
Linnemannia hyalina	UWR_983			+[a, b, c, d]				
Mucor circinelloides	UWR_936		-					
	UWR_984							
	UWR_985			+[a, b]				
Mucor hiemalis	UWR_902	+[a, b, c, d]						
	UWR_903							

	UWR_904	+[a, b, c, d]											
	UWR_905	+[a, b]											
	UWR_986							+[a, b, c, d]					
	UWR_1154												+[a, b, c, d]
Mucor plumbeus	UWR_1095												
	UWR_1096												
Mucor racemosus	UWR_906	+[a, b, c, d]											
Mucor saturninus	UWR_1097											+[a, b]	
Neonectria lugdunensis	UWR_987												
Oidiodendron truncatum	UWR_988												
	UWR_989												
	UWR_990							+[a, b, d]					
	UWR_991							+[a, b, c, d]					
Penicillium atramentosum	UWR_963						+[a]						
	UWR_1068												
	UWR_1098											+[a]	
	UWR_1099											+[a, b, c, d]	
	UWR_1100											+[a]	
	UWR_1101											+[a]	
	UWR_1102											+[b, c]	
	UWR_1103											+[a]	
	UWR_1104											+[a, b, c, d]	
	UWR_1105											+[a]	
Penicillium bialowiezense	UWR_1032								+[b]				
Penicillium chrysogenum	UWR_937					+[a, b, c, d]							
	UWR_938												
	UWR_1106												
	UWR_1107											+[a, b, c, d]	
	UWR_1108												

Penicillium commune	UWR_992						
	UWR_993						
	UWR_1155						
	UWR_1156						
	UWR_907	+[a, b, c, d]					
	UWR_908	+[d]					
	UWR_909						
	UWR_910	+[c]					
	UWR_911	+[a, b]					
	UWR_912	+[b, d]					
	UWR_913	+[a]					
	UWR_1069						
	UWR_1070						
	UWR_1071						
	UWR_1072				+[a, b, c]		
	UWR_1109					+[b, c, d]	
	UWR_1110						
	UWR_1111					+[b]	
	UWR_1112					+[a]	
	UWR_1113					+[d]	
	UWR_1114					+[a, b, c, d]	
	UWR_1115					+[a, b, c, d]	
	UWR_1157						+[b, c, d]
Penicillium corylophilum	UWR_1033						
Penicillium crustosum	UWR_924						
	UWR_939						
	UWR_964		+[b]				
	UWR_965						
	UWR_1052						
	UWR_1053						
	UWR_1054						
	UWR_1055			+[a, b, c, d]			
	UWR_1073				+[a, c]		
	UWR_1074				+[b]		

	UWR_1116												
	UWR_1117											+[a, b, c, d]	
	UWR_1118											+[a, b, c, d]	
	UWR_1119												
	UWR_1120												
	UWR_1121											+[a, b, c, d]	
	UWR_1122											+[a, b, c, d]	
	UWR_1158												+[d]
	UWR_1159												+[b]
	UWR_1160												+[b]
Penicillium expansum	UWR_1161												+[c]
Penicillium griseofulvum	UWR_1123												
Penicillium hordei	UWR_1124											+[b]	
	UWR_966						+[d]						
	UWR_1075										+[a]		
	UWR_1076												
	UWR_1125											+[b, c]	
	UWR_1126											+[a, b, c]	
Penicillium magnielliptisporum	UWR_1127											+[a, b]	
	UWR_1128											+[a]	
	UWR_1129											+[a]	
	UWR_1130											+[a, b]	
	UWR_1131											+[b]	
	UWR_1132											+[a, b]	
Penicillium paneum	UWR_1133												
	UWR_914												
	UWR_915												
	UWR_916												
Penicillium polonicum	UWR_917	+[a, b, c, d]											
	UWR_918	+[c]											
	UWR_919	+[a, b, c, d]											

	UWR_940		+[a, b, c, d]			
Penicillium rubens	UWR_1077					
	UWR_1134			+[a]		
	UWR_1162					
	UWR_1135					
	UWR_1163					+[d]
Penicillium simplicissimum	UWR_994					
	UWR_995					
	UWR_996					
Penicillium solitum	UWR_925					
	UWR_1136				+[a, b, c]	
	UWR_1137				+[a, b, c, d]	
Penicillium speluncae	UWR_1138				+[a, b, c, d]	
Phoma herbarum	UWR_1078					
Pseudopithomyces chartarum	UWR_1164					
Preussia typharum	UWR_1139					
Pseudeurotium zonatum	UWR_997					
Pseudohumicola subspiralis	UWR_998					
Pseudogymnoascus pannorum	UWR_926	+[b, c]				
	UWR_927					
	UWR_928					
	UWR_929	+[d]				
	UWR_930	+[a ,c, d]				
	UWR_931	+[b, c]				
	UWR_932	+[b]				
	UWR_933					
	UWR_934	+[b]				
	UWR_935					
	UWR_941		+[a]			
	UWR_942		+[a, b, c, d]			
	UWR_943		-			
	UWR_944		+[b, c, d]			

	UWR_945	+[d]						
	UWR_946	+[a, b, c, d]						
	UWR_947	+[a, b, c, d]						
	UWR_1034				+[b]			
	UWR_1140							+[a, b, c]
	UWR_999							
	UWR_1000							
	UWR_1001							
	UWR_1002			+[a, b, c]				
	UWR_1003			+[c]				
	UWR_1004			+[a, b, c, d]				
	UWR_1005			+[a, b, c, d]				
	UWR_1006			+[a, b, c, d]				
	UWR_1007			+[a, b, c, d]				
	UWR_1008			+[b, c, d]				
Purpureocillium lilacinum	UWR_1079							
Rhodotorula glutinis	UWR_1080						+[a, b, c, d]	
Rhodotorula mucilaginosa	UWR_1056					+[a, b]		
	UWR_1081						+[a, b]	
Saccharomyces cerevisiae	UWR_1082							
Sarocladium strictum	UWR_1009							
Scedosporium aurantiacum	UWR_1010							
	UWR_1011							
	UWR_1012							
Scopulariopsis brevicaulis	UWR_1013							
	UWR_1165							
Scopulariopsis candida	UWR_1141							
	UWR_1014			+[b, c, d]				
Talaromyces flavus	UWR_1015							
	UWR_1016							

Trichocladium griseum	UWR_1017											
	UWR_1018											
Venustampulla parva	UWR_1142											+[a, b, c]
Yarrowia alimentaria	UWR_1035											
	UWR_1036											
	UWR_1037								+[a, b]			
	UWR_1038								+[a, b, c, d]			
	UWR_1039								-			
	UWR_1040								+[b, d]			
	UWR_1041								+[a, b]			
Yarrowia bubula	UWR_967						+[a]					
Yarrowia deformans	UWR_968											
	UWR_969						+[a, b, c, d]					
	UWR_970						+[a, b, c, d]					
	UWR_1019							+[a, b, c, d]				
	UWR_1020							+[a, b, c, d]				
	UWR_1021							+[a, b]				
Yarrowia galli	UWR_1083										+[a, b]	
Yarrowia lipolytica	UWR_920	+[a, b, c, d]										
	UWR_921											
	UWR_922		+[a, b, c, d]									
	UWR_948											
	UWR_949											
	UWR_950											
	UWR_951											
	UWR_952											
	UWR_953					+[a, b, c]						
	UWR_954											
	UWR_955											
	UWR_956					+[a, b, c, d]						

	UWR_957	+[a, b, c, d]					
	UWR_958						
	UWR_959	+[d]					
	UWR_960	+[a, b, c, d]					
	UWR_971		+[a, b, c, d]				
	UWR_1084				+[a, b, c, d]		
	UWR_1085						
	UWR_1143					+[a, b, c, d]	
	UWR_1144					+[a, b, c, d]	
	UWR_1145					+[a, b, c, d]	
	UWR_1022			+[a, c]			
	UWR_1023			+[c]			
	UWR_1024			+[a, b, c, d]			
	UWR_1025			+[d]			
	UWR_1166						+[a, b, c, d]
	UWR_1167						+[a, b, c, d]
	UWR_1168						+[a, b, c, d]
	UWR_1169						+[a, b, c, d]
Yarrowia parophoni	UWR_1026			+[b, c, d]			
	UWR_1027			+[a, b]			

Table S4 Fungal isolates identified in this study at 25 °C relative to the culture medium. a – YPD $^{pH\ 4.5}$, b – PDA, c – SGA, d – MLNA

Legend: **YPG $^{pH\ 4.5}$** (YPG medium reduced to pH 4.5 and containing chloramphenicol); **PDA** (Potato Dextrose Agar); **SGA** (Sabouraud's Glucose Agar); **MLNA** (Modified Leeming–Notman agar)

Fungi species	Isolate no.	Case number											
		I	II	III	IV	V	VI	VII	VIII	IX	X	XI	XII
Acaulium album	UWR_923			$+^{a, b, c, d}$									
Acaulium caviariforme	UWR_900	$+^{a, b}$											
Akanthomyces muscarius	UWR_972							$+^{a, b, c, d}$					
Alternaria alternata	UWR_1086												
Alternaria infectoria	UWR_1146												$+^{b}$
Apiotrichum dulcitum	UWR_973							$+^{a, b, c, d}$					
Apiotrichum vadense	UWR_974												
Arthrographis kalrae	UWR_975												
Aspergillus flavus	UWR_1147												
Aspergillus niger	UWR_961												
	UWR_1042												
	UWR_1148												$+^{b, c, d}$
Aspergillus versicolor	UWR_976							$+^{a, b, c}$					
Bullera alba	UWR_1057										$+^{b}$		
	UWR_1058												
Candida parapsilosis	UWR_1061										$+^{a}$		
Candida sake	UWR_1087												
Candida zeylanoides	UWR_901												
	UWR_1062												
	UWR_1063										$+^{a, c}$		
	UWR_1064										$+^{b}$		
	UWR_977												
	UWR_1149												
Cladosporium allicinum	UWR_1028												
	UWR_1059										$+^{b, c}$		
Cladosporium cladosporioides	UWR_1043												
	UWR_1044									$+^{c}$			
	UWR_1060										$+^{b, c}$		
Cladosporium halotolerans	UWR_1029												
Cladosporium herbarum	UWR_1150												$+^{b}$

Cladosporium langeronii	UWR_1030												
Cosmospora khandalensis	UWR_978							+[b]					
Debaryomyces hansenii	UWR_962						+[a]						
	UWR_1045												
	UWR_1046									+[a]			
	UWR_1047												
	UWR_1048												
	UWR_1049									+[a, b, c]			
	UWR_1050									+[a, b]			
	UWR_1051									+[a, b]			
	UWR_1065												
	UWR_1088												
	UWR_1089												
	UWR_1090											+[a, b, c, d]	
	UWR_1091											+[a, b, c]	
	UWR_1092												
	UWR_1093											+[a, b, c]	
	UWR_1094											+[a, b, d]	
	UWR_979							+[a, b, c, d]					
	UWR_1151												+[b, c, d]
	UWR_1152												+[a, b, c, d]
	UWR_1153												+[a, b, c, d]
Dialonectria ullevolea	UWR_980							+[b]					
	UWR_981							+[b, c, d]					
Epicoccum nigrum	UWR_1066										+[a, b, c, d]		
	UWR_1067												
Fusarium acuminatum	UWR_1031								+[a, b]				
Fusicolla aquaeductuum	UWR_982												
Linnemannia hyalina	UWR_983							+[a, b, c, d]					
Mucor circinelloides	UWR_936					+[a, b, c, d]							
	UWR_984							+[a, c, d]					
	UWR_985							+[b]					
Mucor hiemalis	UWR_902	+[a, b, c, d]											
	UWR_903	+[a, b]											
	UWR_904												
	UWR_905	+[b, d]											
	UWR_986							+[a, b, c, d]					
	UWR_1154												+[a, b, c, d]
Mucor plumbeus	UWR_1095											+[a, b, c, d]	

	UWR_1096											+[a, b, c, d]	
Mucor racemosus	UWR_906												
Mucor saturninus	UWR_1097											+[a, b]	
Neonectria lugdunensis	UWR_987							+[b]					
Oidiodendron truncatum	UWR_988												
	UWR_989												
	UWR_990							+[a, b]					
	UWR_991							+[a, b, c, d]					
Penicillium atramentosum	UWR_963												
	UWR_1068										+[a]		
	UWR_1098											+[a]	
	UWR_1099												
	UWR_1100												
	UWR_1101												
	UWR_1102											+[a, b]	
	UWR_1103											+[a, b, c, d]	
	UWR_1104											+[a, b]	
	UWR_1105											+[a]	
Penicillium bialowiezense	UWR_1032								+[b]				
Penicillium chrysogenum	UWR_937					+[c]							
	UWR_938					+[a, b, c, d]							
	UWR_1106												
	UWR_1107												
	UWR_1108											+[a, b]	
	UWR_992												
	UWR_993							+[b]					
	UWR_1155												+[b, d]
	UWR_1156												+[a, b, c, d]
Penicillium commune	UWR_907												
	UWR_908	+[a, b, c, d]											
	UWR_909	+[a, b, c, d]											
	UWR_910												
	UWR_911												
	UWR_912												
	UWR_913												
	UWR_1069										+[b, c, d]		
	UWR_1070										+[a, c]		
	UWR_1071										+[b]		
	UWR_1072												

	UWR_1109						+[a, c, d]	
	UWR_1110						+[d]	
	UWR_1111							
	UWR_1112							
	UWR_1113						+[a, b, c, d]	
	UWR_1114							
	UWR_1115						+[a, b, c]	
	UWR_1157							+[c]
Penicillium corylophilum	UWR_1033							
	UWR_924	-						
	UWR_939		+[a, b, c, d]					
	UWR_964							
	UWR_965			+[a, b, c, d]				
	UWR_1052				+[b]			
	UWR_1053				+[a, b, c, d]			
	UWR_1054				+[a, b, c, d]			
	UWR_1055							
	UWR_1073					+[a]		
	UWR_1074					+[a, b, c]		
Penicillium crustosum	UWR_1116							
	UWR_1117						+[c]	
	UWR_1118						+[a, b, c, d]	
	UWR_1119						+[b, c, d]	
	UWR_1120						+[b, c, d]	
	UWR_1121						+[b, c]	
	UWR_1122						+[d]	
	UWR_1158							
	UWR_1159							
	UWR_1160							+[b]
Penicillium expansum	UWR_1161							+[a, d]
Penicillium griseofulvum	UWR_1123						+[b]	
Penicillium hordei	UWR_1124							
	UWR_966							
	UWR_1075							
Penicillium magnielliptisporum	UWR_1076					+[a]		
	UWR_1125							
	UWR_1126							
	UWR_1127							
	UWR_1128							

Penicillium paneum	UWR_1129				+[a]	
	UWR_1130				+[a, b, d]	
	UWR_1131					
	UWR_1132				+[a]	
	UWR_1133					
Penicillium polonicum	UWR_914					
	UWR_915	+[a, b, c, d]				
	UWR_916	+[c, d]				
	UWR_917	+[a, b, c, d]				
	UWR_918					
	UWR_919					
	UWR_940	+[a, b]				
	UWR_1077					
Penicillium rubens	UWR_1134				+[a, b, c, d]	
	UWR_1162					+[b, c, d]
	UWR_1135					
	UWR_1163					+[a, b, c, d]
Penicillium simplicissimum	UWR_994			+[c]		
	UWR_995			+[a, b, c]		
	UWR_996					
Penicillium solitum	UWR_925		+[b]			
	UWR_1136					
	UWR_1137				+[c]	
Penicillium speluncae	UWR_1138					
Phoma herbarum	UWR_1078					
Pseudopithomyces chartarum	UWR_1164					+[b]
Preussia typharum	UWR_1139				+[a, b]	
Pseudeurotium zonatum	UWR_997			+[b]		
Pseudohumicola subspiralis	UWR_998			+[b]		
Pseudogymnoascus pannorum	UWR_926		+[a, b, c, d]			
	UWR_927		+[b]			
	UWR_928		+[a]			
	UWR_929					
	UWR_930					
	UWR_931		+[c]			
	UWR_932		+[a, b, c]			
	UWR_933		+[c]			

	UWR_934	+[b]					
	UWR_935	+[a, b, c, d]					
	UWR_941						
	UWR_942						
	UWR_943		+[a, b, c]				
	UWR_944		+[a, b, c, d]				
	UWR_945		+[a, b, c, d]				
	UWR_946		+[a, b]				
	UWR_947						
	UWR_1034						
	UWR_1140					+[a, b, c, d]	
	UWR_999			+[b, c, d]			
	UWR_1000			+[b]			
	UWR_1001			+[a, b]			
	UWR_1002			+[a, b, c, d]			
	UWR_1003			+[a, b, d]			
	UWR_1004			+[a, b, c, d]			
	UWR_1005			+[a, b, c, d]			
	UWR_1006			+[a, b]			
	UWR_1007			+[a, b, c, d]			
	UWR_1008						
Purpureocillium lilacinum	UWR_1079				+[a, d]		
Rhodotorula glutinis	UWR_1080				+[a, b, c, d]		
Rhodotorula mucilaginosa	UWR_1056						
	UWR_1081				+[a, b, c, d]		
Saccharomyces cerevisiae	UWR_1082						
Sarocladium strictum	UWR_1009			+[a, b]			
Scedosporium aurantiacum	UWR_1010			+[a]			
	UWR_1011			+[c]			
	UWR_1012			+[c]			
Scopulariopsis brevicaulis	UWR_1013			+[a, b, c]			
	UWR_1165						+[a, b, d]
Scopulariopsis candida	UWR_1141					+[a, b, c, d]	
	UWR_1014			+[a, b, c, d]			
Talaromyces flavus	UWR_1015			+[b]			
	UWR_1016			+[b]			
Trichocladium griseum	UWR_1017						
	UWR_1018			+[b]			
Venustampulla parva	UWR_1142						

	UWR_1035								+[a, b, c, d]			
	UWR_1036								+[b, d]			
	UWR_1037								+[a, d]			
Yarrowia alimentaria	UWR_1038											
	UWR_1039								+[a]			
	UWR_1040								+[c]			
	UWR_1041								+[a, b, c]			
Yarrowia bubula	UWR_967						+[a, d]					
	UWR_968						+[a]					
	UWR_969						+[a, d]					
	UWR_970											
Yarrowia deformans	UWR_1019							+[a, b, d]				
	UWR_1020							+[a, b, c, d]				
	UWR_1021											
Yarrowia galli	UWR_1083										+[a, c]	
	UWR_920											
	UWR_921	+[a, b]										
	UWR_922		+[a, b, c, d]									
	UWR_948					+[a, b, d]						
	UWR_949					+[a, b, c, d]						
	UWR_950					+[c]						
	UWR_951					+[a, b, d]						
	UWR_952					+[c]						
	UWR_953					+[a, b]						
	UWR_954					+[a, b]						
	UWR_955					+[a, b, c, d]						
	UWR_956					+[a, b, c, d]						
Yarrowia lipolytica	UWR_957					+[a, b, c, d]						
	UWR_958					+[a]						
	UWR_959					+[a, b, c, d]						
	UWR_960					+[a, b, c, d]						
	UWR_971						+[a, b, c, d]					
	UWR_1084											
	UWR_1085										+[a, c]	
	UWR_1143											
	UWR_1144											+[a, b, c, d]
	UWR_1145											+[a, b, c, d]
	UWR_1022							+[a, b, c, d]				
	UWR_1023											

	UWR_1024							+[a, b, c, d]					
	UWR_1025												
	UWR_1166												+[a, b, c, d]
	UWR_1167												+[a, b, c, d]
	UWR_1168												+[a, b, c, d]
	UWR_1169												+[a, b, c, d]
Yarrowia parophoni	UWR_1026							+[b, c]					
	UWR_1027							+[a, b]					

Table S5 Fungal isolates identified in this study at 37 °C relative to the culture medium. a – YPD $^{pH\ 4.5}$, b – PDA, c – SGA, d – MLNA

Legend: **YPG $^{pH\ 4.5}$** (YPG medium reduced to pH 4.5 and containing chloramphenicol); **PDA** (Potato Dextrose Agar); **SGA** (Sabouraud's Glucose Agar); **MLNA** (Modified Leeming–Notman agar)

**Fungi species**	**Isolate no.**	**Case number**											
		**I**	**II**	**III**	**IV**	**V**	**VI**	**VII**	**VIII**	**IX**	**X**	**XI**	**XII**
Acaulium album	UWR_923			+[a, b, c, d]									
Acaulium caviariforme	UWR_900	+[a, b, d]											
Akanthomyces muscarius	UWR_972												
Alternaria alternata	UWR_1086											+[b, d]	
Alternaria infectoria	UWR_1146												
Apiotrichum dulcitum	UWR_973												
Apiotrichum vadense	UWR_974							+[b]					
Arthrographis kalrae	UWR_975							+[a, d]					
Aspergillus flavus	UWR_1147												+[b]
	UWR_961						+[a]						
Aspergillus niger	UWR_1042									+[a]			
	UWR_1148												+[a, b, c, d]
Aspergillus versicolor	UWR_976							+[b]					
Bullera alba	UWR_1057												
	UWR_1058												
Candida parapsilosis	UWR_1061												
Candida sake	UWR_1087											+[a, b]	
	UWR_901	+[a, b, d]											
Candida zeylanoides	UWR_1062												
	UWR_1063										+[c]		
	UWR_1064										+[a, b, c, d]		

	UWR_977		+[b]					
Cladosporium allicinum	UWR_1149							
	UWR_1028							
	UWR_1059							
Cladosporium cladosporioides	UWR_1043							
	UWR_1044							
	UWR_1060							
Cladosporium halotolerans	UWR_1029			+[c]				
Cladosporium herbarum	UWR_1150							
Cladosporium langeronii	UWR_1030							
Cosmospora khandalensis	UWR_978		+[a, b, c, d]					
Debaryomyces hansenii	UWR_962	+[a]						
	UWR_1045							
	UWR_1046				+[a]			
	UWR_1047							
	UWR_1048							
	UWR_1049				+[a, b, c]			
	UWR_1050				+[a, c]			
	UWR_1051				+[a, b]			
	UWR_1065					+[a, b, c]		
	UWR_1088							
	UWR_1089							
	UWR_1090						+[a, b, c, d]	
	UWR_1091						+[a, b, c, d]	
	UWR_1092							
	UWR_1093						+[a, b, c, d]	
	UWR_1094						+[a, b, c, d]	
	UWR_979		+[a, b, c, d]					
	UWR_1151							+[a, b, d]
	UWR_1152							+[a, b, c, d]
	UWR_1153							+[c, d]
Dialonectria ullevolea	UWR_980							
	UWR_981		+[a]					
Epicoccum nigrum	UWR_1066					+[c]		
	UWR_1067							
Fusarium acuminatum	UWR_1031							
Fusicolla aquaeductuum	UWR_982		+[b]					
Linnemannia hyalina	UWR_983							

Mucor circinelloides	UWR_936					
	UWR_984			+[a, b]		
	UWR_985			+[a, b, c, d]		
Mucor hiemalis	UWR_902	+[a, b, c, d]				
	UWR_903					
	UWR_904					
	UWR_905	+[b, c]				
	UWR_986			+[a, b]		
	UWR_1154					
Mucor plumbeus	UWR_1095					
	UWR_1096				+[b]	
Mucor racemosus	UWR_906					
Mucor saturninus	UWR_1097					
Neonectria lugdunensis	UWR_987					
Oidiodendron truncatum	UWR_988			+[a, b]		
	UWR_989			+[a]		
	UWR_990			+[a, b]		
	UWR_991			+[a, b]		
Penicillium atramentosum	UWR_963					
	UWR_1068					
	UWR_1098				+[a, b]	
	UWR_1099					
	UWR_1100					
	UWR_1101					
	UWR_1102				+[b]	
	UWR_1103				+[b]	
	UWR_1104					
	UWR_1105					
Penicillium bialowiezense	UWR_1032					
Penicillium chrysogenum	UWR_937		+[a, b, c, d]			
	UWR_938		+[c]			
	UWR_1106				+[a, b, c, d]	
	UWR_1107				+[a, b, c, d]	
	UWR_1108				+[a, b, c, d]	
	UWR_992			+[b]		
	UWR_993					
	UWR_1155					+[b, d]
	UWR_1156					+[a, c, d]
Penicillium commune	UWR_907					

	UWR_908	+[a, b, c, d]							
	UWR_909	+[a, b]							
	UWR_910								
	UWR_911								
	UWR_912								
	UWR_913								
	UWR_1069							+[a]	
	UWR_1070								
	UWR_1071							+[a]	
	UWR_1072								
	UWR_1109								
	UWR_1110								+[a]
	UWR_1111								
	UWR_1112								
	UWR_1113								+[a, b, c, d]
	UWR_1114								
	UWR_1115								+[a, b]
	UWR_1157								
Penicillium corylophilum	UWR_1033					+[c]			
	UWR_924		+[b]						
	UWR_939			+[a]					
	UWR_964								
	UWR_965				+[c, d]				
	UWR_1052						+[b]		
	UWR_1053						+[a, b, c, d]		
	UWR_1054						+[a, c, d]		
	UWR_1055						+[c]		
	UWR_1073								
	UWR_1074							+[a, b, c, d]	
Penicillium crustosum	UWR_1116								+[b, c, d]
	UWR_1117								+[a, b, c]
	UWR_1118								
	UWR_1119								+[a, b, c, d]
	UWR_1120								+[a, c]
	UWR_1121								+[b, c]
	UWR_1122								
	UWR_1158								
	UWR_1159								
	UWR_1160								

Penicillium expansum	UWR_1161						
Penicillium griseofulvum	UWR_1123						
Penicillium hordei	UWR_1124						
Penicillium magnielliptisporum	UWR_966						
	UWR_1075						
	UWR_1076						
	UWR_1125						
	UWR_1126						
	UWR_1127						
	UWR_1128						
	UWR_1129						
	UWR_1130					+[b]	
	UWR_1131						
	UWR_1132						
Penicillium paneum	UWR_1133					+[b]	
Penicillium polonicum	UWR_914	+[b, c]					
	UWR_915	+[a, b, c, d]					
	UWR_916	+[b, c, d]					
	UWR_917						
	UWR_918	+[a, b, c, d]					
	UWR_919						
	UWR_940		+[a, b, c, d]				
	UWR_1077						
	UWR_1134					+[a, b, c, d]	
	UWR_1162						+[b]
Penicillium rubens	UWR_1135					+[b, c, d]	
	UWR_1163						+[a, d]
Penicillium simplicissimum	UWR_994			+[c, d]			
	UWR_995			+[b, c, d]			
	UWR_996			+[b, c]			
Penicillium solitum	UWR_925						
	UWR_1136						
	UWR_1137					+[b, c, d]	
Penicillium speluncae	UWR_1138						
Phoma herbarum	UWR_1078				+[c]		
Pseudopithomyces chartarum	UWR_1164						+[a, b]
Preussia typharum	UWR_1139						
Pseudeurotium zonatum	UWR_997			+[b]			

Pseudohumicola subspiralis	UWR_998	+[c]		
Pseudogymnoascus pannorum	UWR_926			
	UWR_927			
	UWR_928			
	UWR_929			
	UWR_930			
	UWR_931			
	UWR_932			
	UWR_933			
	UWR_934			
	UWR_935			
	UWR_941			
	UWR_942			
	UWR_943			
	UWR_944			
	UWR_945			
	UWR_946			
	UWR_947			
	UWR_1034			
	UWR_1140			
	UWR_999			
	UWR_1000			
	UWR_1001			
	UWR_1002			
	UWR_1003			
	UWR_1004			
	UWR_1005			
	UWR_1006			
	UWR_1007			
	UWR_1008			
Purpureocillium lilacinum	UWR_1079			+[c]
Rhodotorula glutinis	UWR_1080			+[a, b, c]
Rhodotorula mucilaginosa	UWR_1056		+[a, b]	
	UWR_1081			+[a, b, c, d]
Saccharomyces cerevisiae	UWR_1082			+[a, b]
Sarocladium strictum	UWR_1009	+[a, b, c]		
Scedosporium aurantiacum	UWR_1010	+[a, d]		
	UWR_1011	+[a]		

Scopulariopsis brevicaulis	UWR_1012												
	UWR_1013							+[b]					
	UWR_1165												+[a, b, c, d]
Scopulariopsis candida	UWR_1141											+[a]	
	UWR_1014							+[a, d]					
Talaromyces flavus	UWR_1015							+[a, c]					
	UWR_1016												
Trichocladium griseum	UWR_1017							+[a]					
	UWR_1018							+[a, b]					
Venustampulla parva	UWR_1142												
Yarrowia alimentaria	UWR_1035								+[b]				
	UWR_1036												
	UWR_1037												
	UWR_1038												
	UWR_1039												
	UWR_1040								+[c]				
	UWR_1041								+[b]				
Yarrowia bubula	UWR_967						+[d]						
Yarrowia deformans	UWR_968						+[a, b, c]						
	UWR_969						+[d]						
	UWR_970												
	UWR_1019							+[a, b, c, d]					
	UWR_1020							+[a, b, c, d]					
	UWR_1021												
Yarrowia galli	UWR_1083										+[b, c]		
Yarrowia lipolytica	UWR_920	+[a, b, c, d]											
	UWR_921	+[a, b, c, d]											
	UWR_922		+[a, b, c, d]										
	UWR_948					+[a]							
	UWR_949					+[a, b, c, d]							
	UWR_950					+[c]							
	UWR_951					+[d]							
	UWR_952					+[c]							
	UWR_953												
	UWR_954												
	UWR_955					+[a, c, d]							
	UWR_956					+[a, c, d]							
	UWR_957					+[a, b, c, d]							
	UWR_958					+[a, c, d]							

	UWR_959	+[a, b, c, d]					
	UWR_960	+[a, b, c, d]					
	UWR_971		+[a, b, c, d]				
	UWR_1084				+[b]		
	UWR_1085				+[a, c]		
	UWR_1143					+[a, b, c, d]	
	UWR_1144					+ [b, c, d]	
	UWR_1145					+[a, b, c, d]	
	UWR_1022			+[a, b, c, d]			
	UWR_1023						
	UWR_1024			+[a, b, c, d]			
	UWR_1025						
	UWR_1166						+[a, b, c, d]
	UWR_1167						+[a, b, c, d]
	UWR_1168						+[a, b, c, d]
	UWR_1169						+[a, b, c, d]
Yarrowia parophoni	UWR_1026			+[a, c]			
	UWR_1027			+[a]			
